# A soybean sodium/hydrogen exchanger GmNHX6 confers plant alkaline salt tolerance by regulating Na^+^/K^+^ homeostasis

**DOI:** 10.3389/fpls.2022.938635

**Published:** 2022-09-20

**Authors:** Ting Jin, Jiaxin An, Huadong Xu, Jie Chen, Lang Pan, Ranran Zhao, Ning Wang, Junyi Gai, Yan Li

**Affiliations:** National Key Laboratory of Crop Genetics and Germplasm Enhancement, Key Laboratory for Biology and Genetic Improvement of Soybean (General, Ministry of Agriculture), National Center for Soybean Improvement, Jiangsu Collaborative Innovation Center for Modern Crop Production, Nanjing Agricultural University, Nanjing, China

**Keywords:** abiotic stress, alkaline salt tolerance, natural variation, promoter, sodium bicarbonate, sodium hydrogen exchanger, soybean

## Abstract

Alkaline soil has a high pH due to carbonate salts and usually causes more detrimental effects on crop growth than saline soil. Sodium hydrogen exchangers (NHXs) are pivotal regulators of cellular Na^+^/K^+^ and pH homeostasis, which is essential for salt tolerance; however, their role in alkaline salt tolerance is largely unknown. Therefore, in this study, we investigated the function of a soybean *NHX* gene, *GmNHX6*, in plant response to alkaline salt stress. *GmNHX6* encodes a Golgi-localized sodium/hydrogen exchanger, and its transcript abundance is more upregulated in alkaline salt tolerant soybean variety in response to NaHCO_3_ stress. Ectopic expression of *GmNHX6* in *Arabidopsis* enhanced alkaline salt tolerance by maintaining high K^+^ content and low Na^+^/K^+^ ratio. Overexpression of *GmNHX6* also improved soybean tolerance to alkaline salt stress. A single nucleotide polymorphism in the promoter region of *NHX6* is associated with the alkaline salt tolerance in soybean germplasm. A superior promoter of *GmNHX6* was isolated from an alkaline salt tolerant soybean variety, which showed stronger activity than the promoter from an alkaline salt sensitive soybean variety in response to alkali stress, by luciferase transient expression assays. Our results suggested soybean *NHX6* gene plays an important role in plant tolerance to alkaline salt stress.

## Introduction

Saline-alkali soils currently account for 20% of irrigated land (Bouras et al., 2022). Land alkalization has become a major and increasingly serious problem in the world. Compared with neutral salt stress, the high pH environment in alkaline soil destroys soil structure and affects the absorption of essential elements such as phosphorus and iron by plants (Shi and Wang, 2005). Therefore, alkaline salt causes more serious effects than neutral salt stress on plants. Soil alkalization can reduce soil osmotic potential, and cause ion imbalance, disrupt physiological processes, inhibit the growth and development of plants, leading to a serious decline in the yield and quality, and even the death of plants (Yang et al., 2011). Saline-alkali stress leads to leaf wilting and chlorosis (Sun et al., 2021), fewer pods, seeds and lower 100-seed weight (He et al., 2020), inhibited nodule development (He et al., 2021) and other phenomena (Chen et al., 2018) of soybean, resulting in soybean growth retardation, and eventually lead to a serious decline in soybean yield (He et al., 2020). Plants can effectively reduce the damage caused by salt-alkali stress through ion selective absorption, compartmentalization and scavenging reactive oxygen species, etc. (Zhu, 2001; Zhang et al., 2019a). Sodium hydrogen exchanger (NHXs) are integral membrane proteins residing in the plasma membrane, endosome (Dragwidge et al., 2019), and vacuole (Sellamuthu et al., 2020), which belong to the monovalent cation/proton antiporter family (Bassil et al., 2011b). NHXs play important roles in regulating of cellular ion homeostasis (Bassil et al., 2019), pH (Reguera et al., 2015), vesicle trafficking (Bassil et al., 2019), protein transport (Dragwidge et al., 2019), auxin transport (Zhang et al., 2020a), salt tolerance (Long et al., 2020), cell turgor and expansion (Walker et al., 1996), as well as growth and development (Bassil et al., 2019) in many species ranging from bacteria to human. In plants, NHX utilizes an H^+^ electrochemical gradient established by (H^+^)-ATPase (in the plasma membrane) and (H^+^)-PPase (in vacuoles) to allow Na^+^ or K^+^ exchange for H^+^, to maintain pH and ion homeostasis (Rodríguez-Rosales et al., 2009; Bassil et al., 2012).

In *Arabidopsis thaliana*, the NHX exchanger family contains eight members which are divided into three subclasses based on their subcellular localizations: vacuolar (AtNHX1-AtNHX4), endosomal (AtNHX5 and AtNHX6), and plasma membrane (AtNHX7/SOS1and AtNHX8) localized exchangers (Reguera et al., 2014; Qiu, 2016a). Plasma membrane NHXs (AtNHX7/SOS1and AtNHX8) are required for Na^+^, K^+^, and pH homeostasis, and play an important role in salt tolerance (An et al., 2007; Oh et al., 2010). Vacuolar membrane NHXs (AtNHX1-AtNHX4) are critical for vacuolar pH and K^+^ homeostasis (Barragán et al., 2012), salt and drought stress responses (Moghaieb et al., 2014; Zhang et al., 2019b), osmotic adjustment (Quintero et al., 2000), flower development (Bassil et al., 2011b), as well as plant growth and development (Qiu, 2016a). AtNHX5 and AtNHX6 localize to the Golgi and trans-Golgi network (TGN), and are important for plant growth and response to salt stress (Bassil et al., 2011b), maintaining ion and pH homeostasis (Wang et al., 2015), protein transport (Qiu, 2016b), plant growth and development (Lv et al., 2020), and seedling growth (Zhang et al., 2020b). The *Arabidopsis nhx5 nhx6* double mutant showed reduced growth, smaller and fewer cells, smaller rosettes and shorter seedlings, late flowering, and increased sensitivity to salinity (Bassil et al., 2011a). The *nhx5 nhx6 syp22* triple mutant had short siliques and low seed setting rate, but larger seeds. In addition, the triple mutant had numerous smaller protein storage vacuoles (PSVs) and accumulated precursors of seed storage proteins, suggesting that *AtNHX5* and *AtNHX6* play important roles in seed production, protein trafficking and PSV biogenesis (Wu et al., 2016). A recent report suggests that NHX5 and NHX6 might regulate auxin transport through endoplasmic reticulum (Fan et al., 2018).

There are also few studies on NHX5 or NHX6 from other plant species in addition to *Arabidopsis*. Overexpression of *PdNHX6* from *Phoenix dactylifera* in *Arabidopsis* plants enhanced salt tolerance, retained higher chlorophyll and water content, maintained a balanced Na^+^/K^+^ ratio, and increased seed germination under salinity when compared to the wild-type plants (Al-Harrasi et al., 2020). *AoNHX6* from *Avicennia officinalis* showed high expression levels in the roots, and complementation with *AoNHX6* improved the tolerance of yeast mutants and *Arabidopsis* mutants to both NaCl and KCl stress (Krishnamurthy et al., 2019). The ectopic expression of endosomal-type *MnNHX6* from *Morus notabilis* in *Arabidopsis* and *nhx1* yeast mutant can greatly enhance their salt tolerance compared with vacuolar-type MnNHXs (Cao et al., 2020). Up to now, the role of *NHX* genes in soybean tolerance to salt-alkali stress is largely unknown, except that *GmNHX5* is found regulating salt tolerance in a recent study (Sun et al., 2021). Alkaline soils cause damage to plants not only through salt stress, but also through high pH (Shi and Wang, 2005), therefore, alkali stress is more serious than salt stress, while the role of *NHX* genes in alkali tolerance of soybean remains unclear.

In our previous transcriptomic study (unpublished) using an alkaline salt tolerant wild soybean variety, we identified an alkali-responsive gene (corresponding to Glyma.09G018200 in the reference genome of soybean variety Williams 82), which encodes a sodium/hydrogen exchanger, and is designated as *GmNHX6*. Here we investigated the role of *GmNHX6* in alkali stress by comparing the relative expression level of *GmNHX6* gene in alkaline salt tolerant and sensitive soybean varieties, and study the function of this gene in transgenic soybean composite plants and *Arabidopsis*. The sequence variation in *GmNHX6* and its association with alkaline salt tolerance was also analyzed, to gain a better understanding of its role in alkaline salt tolerance. The possible molecular mechanism of *GmNHX6* in response to alkaline salt stress was investigated by the content of Na^+^, K^+^, the Na^+^/K^+^ ratio, and promoter luciferase (LUC) assay. This study aims to provide new insights into the role of *GmNHX6* in soybean tolerance to alkaline salt stress and the possible underlying mechanisms.

## Materials and methods

### Soybean accessions and sodic tolerance rating

A total of 60 soybean accessions (Supplementary Table 1), were obtained from the National Center for Soybean Improvement (Nanjing, China). Twelve seeds of each soybean accession were germinated in plastic pots (φ10 × 8 cm) filled with sterile nutrient soil, and irrigated with 1/2 Hoagland nutrient solution form bottom, at 28°C (day)/24°C (night) with 14 h (light)/10 h (dark) photoperiod. Fifteen pots were placed in a plastic tray (55 cm × 36 cm × 8 cm) containing 2 L fresh 1/2 Hoagland nutrient solution (pH ≈ 6.5), and the solution was changed every 2 days. After emergence, the seedings were thinned to four plants in each pot. When the second trifoliolate leaves appeared (12-day-old soybean seedlings), plants were treated with 1/2 Hoagland solution containing 0 or 90 mM NaHCO_3_. Sixteen days after treatment, the alkaline salt tolerance was determined by Sodic Tolerance Rating (STR) according to the previously published method (Tuyen et al., 2010).

### RNA extraction and gene expression analysis

RNA-seq data from different soybean tissues were downloaded from Soybase.^1^ To experimentally determine the expression of *GmNHX6*, tissues were collected from soybean plants grown in vermiculite in greenhouse with controlled temperature (day/night, 28°C/24°C) and light cycle (day/night, 14/10 h). *Arabidopsis thaliana* plants were grown under controlled temperatures of day/night as 22/22°C and light cycle of day/night for 16/8 h. Fresh tissues were ground and extracted for total RNA using an RNAprep Pure Plant Kit (Tiangen Biotech, China). The gene specific primers were designed using the Primer Premier 5 software^2^ and listed in Supplementary Table 2. The cDNA was synthesized by the PrimeScript™ 1st Strand cDNA Synthesis Kit (TaKaRa, Japan). The qRT-PCR was conducted using SYBR Premix ExTaq™ II Mix (TaKaRa, Japan) on a Roche 480 Real-time detection system (Roche Diagnostics, Switzerland) according to the manufacturers’ instructions. Each experiment was performed in triplicates. Transcript levels in soybean plants were calculated in relative to the reference gene *GmUKN1* (Guan et al., 2014), using the 2^-△△CT^ methods (Livak and Schmittgen, 2001). The expression of *GmUKN1* is stable across all samples in this study (Supplementary Table 3). The qRT-PCR of *GmNHX6* gene in transgenic *A. thaliana* was conducted using the OE-2 line (with lowest expression level of *GmNHX6*) as the control (relative expression = 1) and *AtACTIN7* (Miao et al., 2020) as the reference gene. Data was collected from three biological replicates. The amplification efficiencies (E) of primer pairs were estimated (Supplementary Table 2) by qRT-PCR using 1:10, 1:20, 1:40, 1:80, and 1:160 dilutions of cDNA templates, according to the equation: E = [10^−1/slope^] − 1 (Pfaffl, 2001).

### Sequence alignment, phylogenetic analysis, and sequence analysis

The full sequences of GmNHX6 and other NHXs proteins obtained from Phytozome,^3^ were used for multiple sequence alignments by ClustalW2.^4^ The unrooted phylogenetic tree was then constructed using MEGA 6.0 (Tamura et al., 2013) based on the Maximum Likelihood (ML) algorithm with 1,000 bootstraps. The putative promoter sequence of 2000-bp upstream *GmNHX6* was analyzed using the PlantCARE.^5^ Protein transmembrane topology and signal peptides were predicted from amino acid sequences using Protter database.^6^

### Subcellular localization of GmNHX6

For subcellular localization of GmNHX6 protein, the open reading frame (ORF) of *GmNHX6* was amplified by PCR using specific primers (Supplementary Table 2). The PCR product was then introduced into the pAN580 vector, which contains a green florescence protein (GFP) reporter gene, under the drive of CaMV 35S promoter for N-terminal GFP fusion (Zhang et al., 2016), using the ClonExpress II One Step Cloning Kit (Vazyme, China) according to the manufacturer’s protocol. To further determine the specific subcellular localization of GmNHX6, the fluorescent marker protein, Man1-mCherry, which is characteristic for the *cis*-Golgi (Tse et al., 2004), was co-expressed with GmNHX6. The transient expression of above proteins in *Arabidopsis* mesophyll protoplasts was performed following the published methods (Wu et al., 2009). Confocal imaging analysis was performed using a laser scanning microscope (Zeiss LSM780 META, Jena, Germany).

### Obtaining and phenotyping of transgenic soybean composite plants

The coding sequence of *GmNHX6* from soybean accession M8206 was cloned into pBinGFP_4_ vector under the control of CaMV 35S promoter. The construct containing 35S:*GmNHX6* and the empty vector pBinGFP_4_, were separately transformed into *Agrobacterium rhizogenes* strain K599 (Kereszt et al., 2007), then used to infect soybean hypocotyls of an alkaline salt sensitive soybean variety Tianlong 1, to obtain transgenic soybean composite plants, according to the previously described method (Kereszt et al., 2007). After 20 days of plant growth, the green fluorescence signals of GFP in positive hairy roots were identified at 488 nm wavelength using a stereoscopic fluorescence microscope (Mshot, China), and the non-transgenic roots were cut-off. The transgenic soybean composite plants were treated with 1/2 Hoagland solution containing 0 or 90 mM NaHCO_3_ for 7 days, then the Soil and Plant Analysis Development (SPAD) value for chlorophyll content (Pérez-Patricio et al., 2018) and leaf relative water content (LRWC) (Zhang and Zhang, 2021) were measured. The SPAD values of the second, third and fourth trifoliolate leaves were recorded 10 times each by the Chlorophyll Meter SPAD-502 Plus (Konica Minolta, Tokyo, Japan), and the average value was calculated as the SPAD value of the plant. To measure LRWC, the fresh weight (FW) of the second, third and fourth trifoliolate leaves was recorded, and then the leaves were incubated in sterile water at room temperature for 12 h, and the turgid fresh weight (TFW) was measured. The fully turgid leaves were then dried at 80°C for 72 h and the dry weight (DW) was recorded. LRWC was measured according to the following equation: LRWC = (FW – DW)/(TFW – DW) × 100%. Three biological replications were performed and five independent transgenic plants (*n* = 3 × 5 = 15) were measured for each repeat.

### Functional analysis of *GmNHX6* in *Arabidopsis thaliana*

The coding sequence of *GmNHX6* from soybean accession M8206 was cloned into pCAMBIA3301 vector under the control of CaMV 35S promoter. The pCAMBIA3301-35S:*GmNHX6* vector was introduced into the *A. tumefaciens* strain EHA105 for *A. thaliana* transformation using Col-0 wild-type (WT) plants by the floral dipping method (Clough and Bent, 1998). Transgenic plants were screened based on 20 mg L^−1^ glufosinate (PhytoTech, United States) resistance (Ren et al., 2009). The homozygous T_3_ generation plants were subjected to *GmNHX6* gene expression analyses and phenotypic evaluation. The *Arabidopsis nhx6* T-DNA insertion mutant (stock, *SALK-100042C*, *nhx6*, Col-0 background) used in this study was obtained from the *Arabidopsis* Biological Resource Center.^7^

To measure the seed germination rate of *A. thaliana*, 36 stratified seeds for each of three independent *GmNHX6*-overexpressed lines (OE-1, OE-9, and OE-18), as well WT and *nhx6* mutant, were plated on 1/2 MS agar medium, or the media supplemented with 150 mM NaCl (Zheng et al., 2021) or 8 mM NaHCO_3_ (Zhu et al., 2011), and then placed in a growth chamber under the long-day growth conditions (16 h light/8 h dark cycle at 22°C ± 2°C). The rates of cotyledon greening were measured as seed germination rates after 7 days. To measure the root length and fresh weight of *Arabidopsis* seedlings, the sterilized seeds of *Arabidopsis* were vertically grown on 1/2 MS agar medium for 7 days, and then the seedlings were transplanted to fresh 1/2 MS agar medium, or media supplemented with 150 mM NaCl (Zheng et al., 2021) or 8 mM NaHCO_3_ (Zhu et al., 2011) for another 10 days. The root length was analyzed using Image J software.^8^

### Determination of ion content

Seven-day-old *Arabidopsis* seedlings were transplanted to fresh 1/2 MS agar medium, or media supplemented with 150 mM NaCl or 8 mM NaHCO_3_ for another 10 days. Then the seedlings were used for Na^+^ and K^+^ content determination. To determine the ion content, plants were rinsed thoroughly in deionized water, separated into roots and shoots, inactivated at 105°C for 2 h, and oven dried at 80°C until they attained a constant mass. The 0.1 g dry material was mineralized using 2 ml 10% HNO_3_. Then, the microwave digestion system ETHOS T (Milestone, Italy)was used for ion extraction, and the ion contents in the supernatants were analyzed using an Optima 8,000 ICP-OES DV Spectrometer (PerkinElmer, United States) according to the manufacturer’s instructions (Munns et al., 2012). Three biological replicates were performed.

### Analysis of sequence variation and regional association study

Nucleotide sequences were downloaded from phytozome v10 (see Footnote 3), and the 2000-bp upstream of the start codon ATG was considered as the promoter region. The full-length coding sequences were amplified using soybean cDNA as template, and the promoter regions were amplified using soybean genomic DNA as template. The amplicons were sequenced at GenScript (Nanjing, China), and sequence variations among soybean accessions were analyzed. Regional association study was performed using TASSEL 5.0 software (Bradbury et al., 2007). The threshold for a significant association was determined using the previously published method (Yang et al., 2014; Wang et al., 2020), which is *p* < 3.3 × 10^−2^ (1/*n*, where n is the number of markers) in this study.

### Promoter-LUC assays in tobacco leaves

The promoter activities were analyzed by promoter-LUC transient expression assays in tobacco (*Nicotiana benthamiana*) leaves, according to the procedures described previously (Jin et al., 2021). For vector constructs, the full-length 2-kb promoter regions of M8206 and ZY were amplified, respectively, and the amplicons were inserted into pGreenII0800-LUC to obtain different promoter-LUC vectors. Above constructs with LUC as a reporter, Pro(ZY)-LUC, Pro(M)-LUC, were transformed into *A. tumefaciens* strain GV3101 that carries pSoup-19, and introduced into tobacco leaves by infiltration. Then, the tobacco seedlings were transferred to 0 or 100 mM NaHCO_3_ for 16 h. LUC activity was observed with an *in vivo* plant imaging system (Berthold LB 985, Germany).

### Statistical analyses

For qRT–PCR analyses, at least four individual plants were pooled per tissue sample, and at least three qRT–PCR reactions (technical replicates) with at least three biological replicates were performed. For phenotypic evaluation, at least 12 individual plants were analyzed per accession, and the exact numbers of individuals (*n*) are presented in all figure legends. Differences between groups or genotypes were analyzed using two-sided Wilcoxon test, Duncan’s multiple range test or Student’s *t*-test by R software and SAS 9.2 (SAS Institute Inc., Cary, NC, United States).

## Results

### GmNHX6 encodes a Golgi-localized sodium/hydrogen exchanger

**Figure 1:**
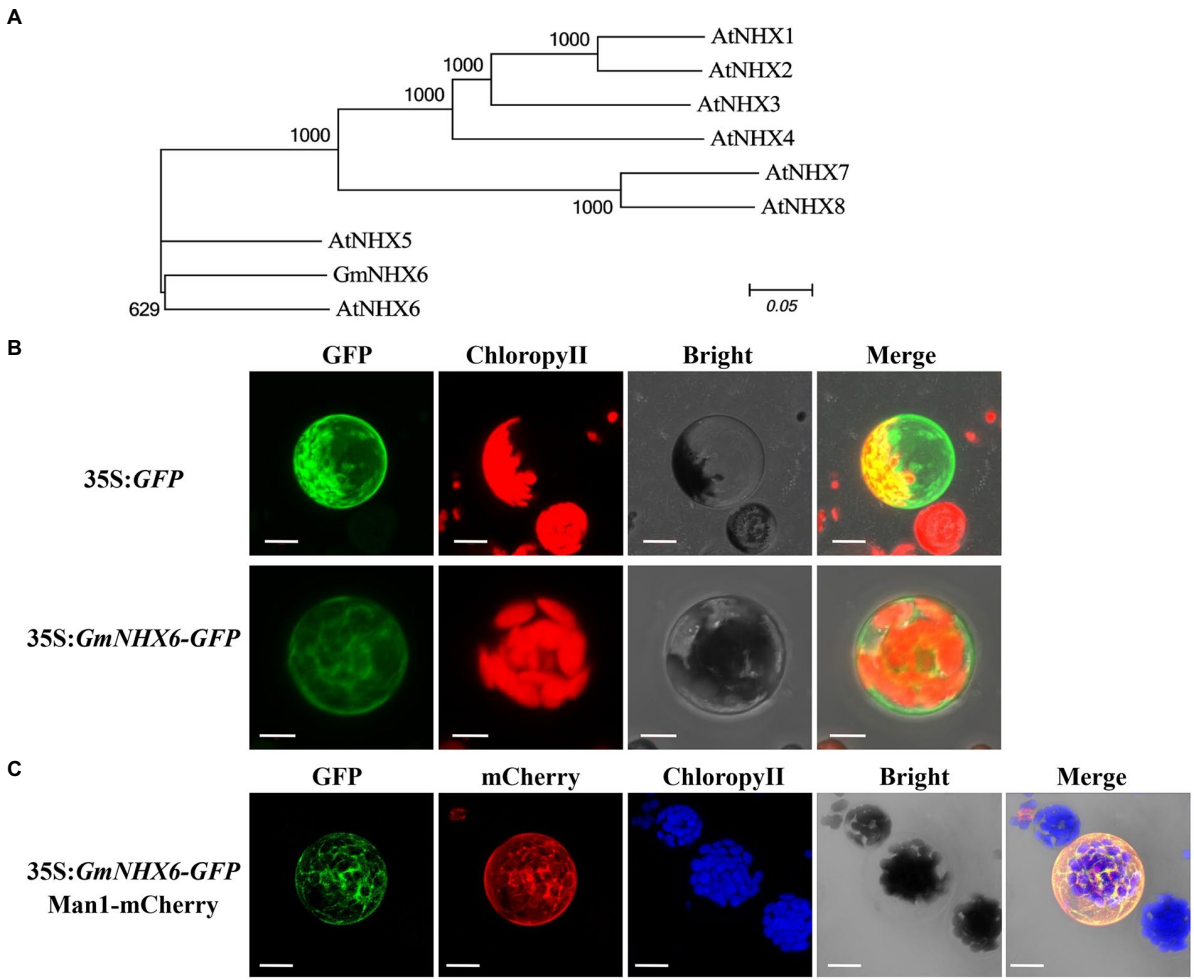
Phylogenetic analysis and subcellular localization of GmNHX6. **(A)** Phylogenetic analysis of GmNHX6 (XP_028181010.1) from soybean *Glycine max* and NHX proteins from *Arabidopsis thaliana* including AtNHX1 (NP_198067.1), AtNHX2 (NP_187154.1), AtNHX3 (NP_200358.1), AtNHX4 (NP_187288.2), AtNHX5 (NP_175839.2), AtNHX6 (NP_178079.2), AtNHX7 (NP_178307.2), AtNHX8 (NP_172918.2). The sequences of NHX proteins were downloaded from the Phytozome database. Unrooted phylogenetic tree was constructed using MEGA6.0 based on the Maximum Likelihood algorithm with 1,000 bootstraps. **(B)** Subcellular localization of GmNHX6. GFP protein and GmNHX6-GFP fusion protein expressed under the control of CaMV 35S promoter in *Arabidopsis* protoplasts were observed under a confocal microscope. Bar, 10 μm. **(C)** Images of *Arabidopsis* mesophyll protoplasts co-expressing GmNHX6-GFP fusion protein and a mCherry Golgi apparatus marker (Man1-mCherry). Bars = 10 μm.

*GmNHX6* gene encodes a sodium/hydrogen exchanger consisting of 534 amino acids. Phylogenetic analyses of GmNHX6 with NHX proteins in *Arabidopsis* showed that GmNHX6 is closely related with AtNHX6 (Figure 1A). Prediction of GmNHX6 transmembrane topology displays 12 transmembrane domains (Supplementary Figure 1), sharing high similarities with AtNHX6. GmNHX6 contains four conserved acidic residues (Al-Harrasi et al., 2020) in transmembrane domains (Supplementary Figure 2), which were previously proven to be essential for K^+^ transport (Wang et al., 2015). To determine the subcellular localization of GmNHX6, the *GmNHX6* gene was expressed in fusion with the *GFP* reporter gene, in the pAN580-GFP expression vector. The constructed vector and empty control vector were transferred to the protoplast of *Arabidopsis*, and the location of GFP or its fusion protein was observed. We found that, GFP itself was distributed evenly in the cytoplasm and the nucleus, whereas the GmNHX6–GFP fusion protein was mainly localized to the cytoplasm and to punctate compartments in the cytosol (Figure 1B). To determine the nature of these punctate compartments, we co-expressed GmNHX6–GFP and the fluorescent marker characteristic for the Golgi, Man1-mCherry (Tse et al., 2004). The co-expression results showed that GmNHX6-GFP fusion protein was mainly located in the Golgi (Figure 1C).

### The expression of *GmNHX6* is more upregulated in alkaline salt tolerant soybean in response to NaHCO_3_ stress

**Figure 2:**
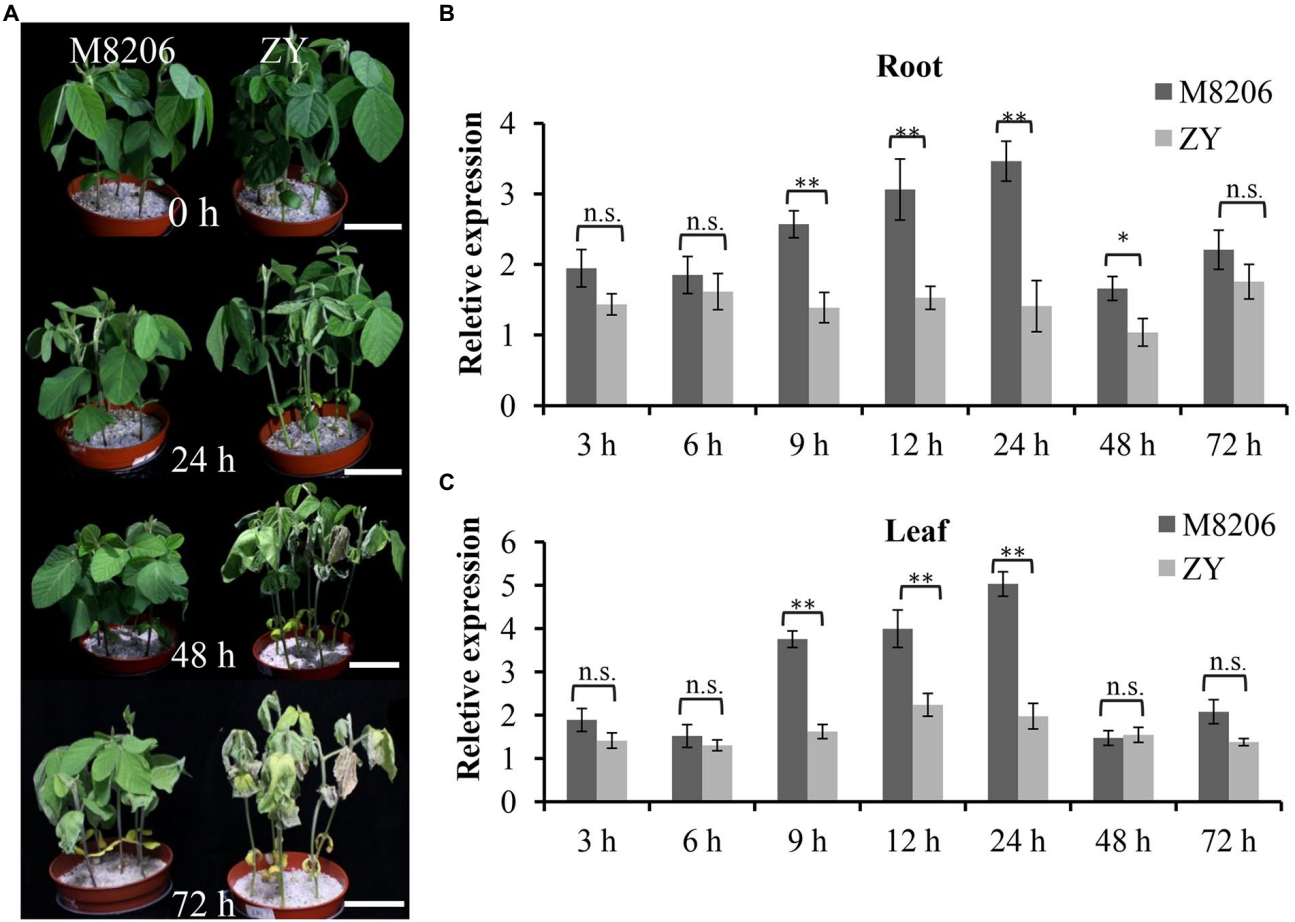
Phenotype and relative expression of *GmNHX6* in two soybean varieties in response to alkali stress. **(A)** Phenotypes of two soybean varieties at 0, 24, 48, and 72 h after 90 mM NaHCO_3_ treatment. The 12-day-old seedlings were subjected to treatment. Bar = 5 cm. **(B,C)** Relative expression of *GmNHX6* in response to alkali stress in roots and leaves, respectively. Soybean seedlings were treated with 0 or 90 mM NaHCO_3_ for 3, 6, 9, 12, 24, 48, and 72 h. Roots and leaves receiving 0 mM NaHCO_3_ treatment at each time point were used as controls. Data represents the mean ± standard deviation of three biological replications with three repeats within each replication (n = 3 × 3 = 9). Differences between two soybean varieties were evaluated using the two-tailed Student’s *t-*tests (n.s., not significant; ^*^p < 0.05; ^**^p < 0.01).

The phenotypic difference between two soybean varieties of M8206 (alkaline salt tolerant) and ZY (alkaline salt sensitive) was obvious under 90 mM NaHCO_3_ stress (Figure 2A): ZY already showed wilted leaves at 24 h, but the leaves of M8206 were still normal. The leaves of ZY became yellow and dry out at 48 h and more severe at 72 h, while the leaves of M8206 did not show obvious wilting at 48 h until 72 h. The expression patterns of *GmNHX6* in *G. max* (see Footnote 1) showed that the transcript abundance of *GmNHX6* was higher in the young_leaf and root than the other organs (Supplementary Figure 3). The relative expression levels of *GmNHX6* in response to alkaline salt stress (90 mM NaHCO_3_) in two soybean varieties were investigated using qRT-PCR. *GmNHX6* gene expression was induced by 90 mM NaHCO_3_ treatment in the roots and leaves of both soybean varieties (Figures 2B,C). The relative expression level of *GmNHX6* gene in the roots of M8206 (alkaline salt tolerant) showed significantly greater increase at 9, 12, 24, and 48 h after 90 mM NaHCO_3_ treatment than the alkaline salt sensitive soybean variety ZY (Figure 2B), and the relative expression level of *GmNHX6* gene in the leaves of M8206 showed significantly greater increase at 9, 12, and 24 h in response to NaHCO_3_ treatment than ZY (Figure 2C). There was no significant difference in *GmNHX6* gene expression between two soybean varieties under normal condition (Supplementary Figure 4). These results suggested that *GmNHX6* might play an important role in soybean response to alkaline salt stress.

### Overexpression of *GmNHX6* improved soybean tolerance to NaHCO_3_ stress

**Figure 3:**
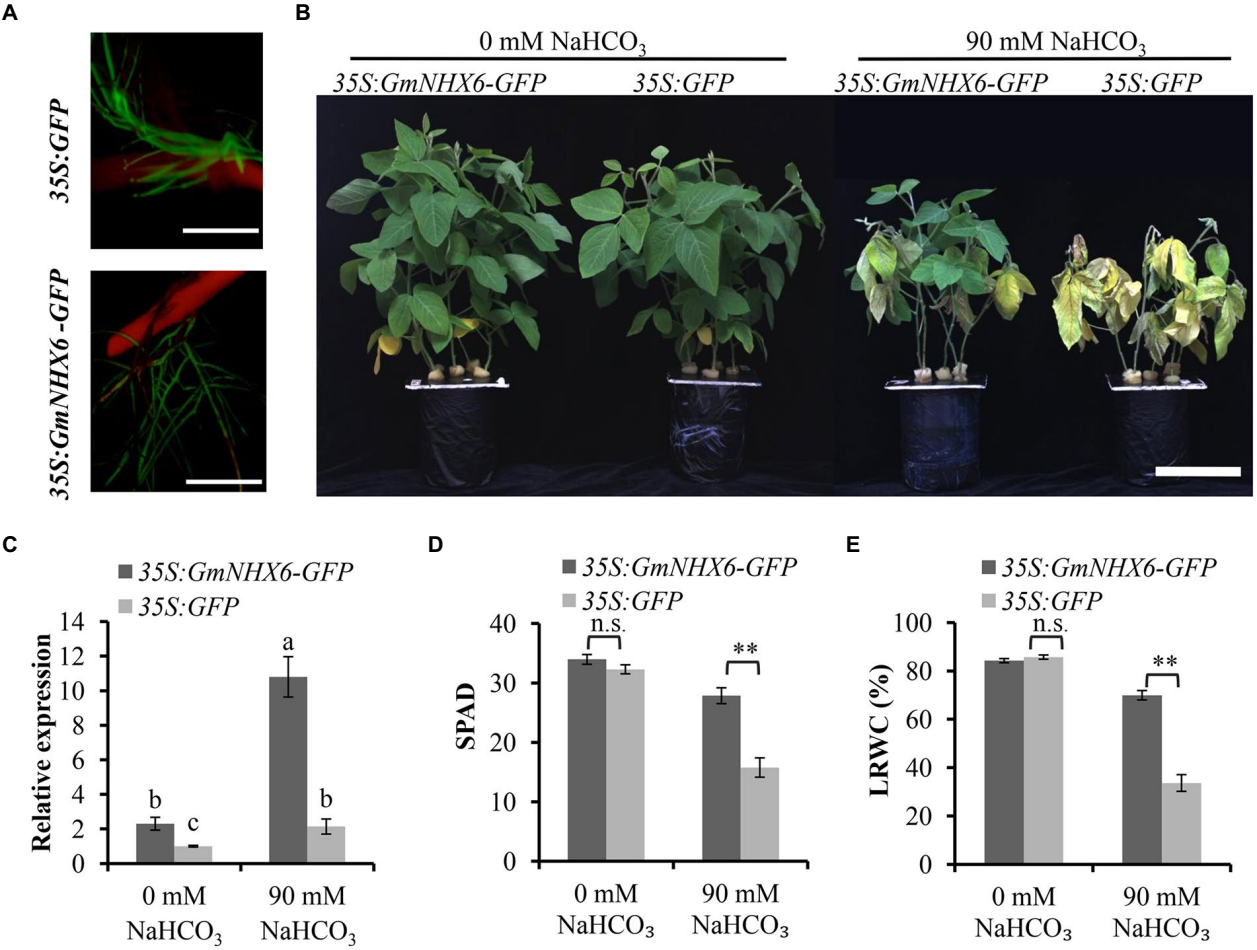
Alkaline salt tolerance analyses of transgenic soybean composite plants. **(A)** Identification of positive transgenic soybean lines by green fluorescence using a stereoscopic fluorescence microscope. Bar = 1 cm. **(B)** Representative phenotype of transgenic soybean composite plants (25-day-old) under 0 mM or 90 mM NaHCO_3_ for 7 days. Bar = 12 cm. **(C)** Relative expression of *GmNHX6* gene in the roots of soybean composite plants at 24 h after 0 or 90 mM NaHCO_3_ treatment by qRT-PCR. For relative expression calculation, the sample from soybean composite plants with empty vector (35S:*GFP*) under 0 mM NaHCO_3_ was used as control and *GmUKN1* was the reference gene. Data represents the mean ± standard deviation of three biological replications with three repeats within each replication (*n* = 3 × 3 = 9). Bars with the same letter in lowercase above bars indicate no significant difference according to Duncan’s multiple range test at 0.05 level. **(D,E)** The Soil and Plant Analysis Development (SPAD) value for chlorophyll content, and leaf relative water content (LRWC) of transgenic soybean composite plants under 0 mM or 90 mM NaHCO_3_ for 7 days, respectively. Data represents the mean ± standard deviation of three biological replications and each repeat contained five independent transgenic plants for each genotype (*n* = 3 × 5 = 15). Soybean variety of “TianLong1” was used. Differences were evaluated using the two-tailed Student’s *t*-tests (n.s., not significant; ^**^p < 0.01).

Since a greater increase in the expression level of *GmNHX6* was observed in alkaline salt tolerant soybean variety than the sensitive variety after NaHCO_3_ treatment, we next tested whether overexpression of *GmNHX6* could improve soybean tolerance to alkaline salt stress or not. The coding region of *GmNHX6* gene was expressed in fusion with *GFP* (while the empty vector 35S:*GFP* was used as control), and transformed into soybean hypocotyls to obtain transgenic composite plants, in the genetic background of an alkaline salt sensitive soybean variety Tianlong 1. The positive transgenic composite soybean plants were identified through GFP fluorescence signal in roots (Figure 3A). Under normal condition (0 mM NaHCO_3_), all soybean composite plants grew well, with no obvious difference (Figure 3B; Supplementary Figures 5A,E,I). When the transgenic soybean composite plants were treated with 90 mM NaHCO_3_ for 7 days, the 35S:*GFP*-transformed soybean composite plants showed obviously inhibited growth, leaf wilting and chlorosis, while the 35S:*GmNHX6-GFP-*transformed composite plants had much less damage (Figure 3B; Supplementary Figures 5A,E,I). The expression level of *GmNHX6* gene was higher in 35S:*GmNHX6-GFP-*transformed soybean plants than the 35S:*GFP*-transformed plants either under 0 or 90 mM NaHCO_3_, and *GmNHX6* gene expression was significantly upregulated at 24 h post 90 mM NaHCO_3_ treatment in 35S:*GmNHX6-GFP-*transformed soybean hairy roots (Figure 3C; Supplementary Figures 5B,F,J). The average SPAD value for chlorophyll content (Figure 3D; Supplementary Figures 5C,G,K) and the leaf relative water content (LRWC; Figure 3E; Supplementary Figures 5D,H,L) of 35S:*GmNHX6-GFP-*transformed soybean composite plants were significantly higher than those of the control plants transformed by the 35S:*GFP* under 90 mM NaHCO_3_ treatment. No significant differences were found in SPAD and LRWC between 35S:*GmNHX6-GFP-*transformed plants and 35S:*GFP*-transformed plants under normal condition (0 mM NaHCO_3_). These results demonstrated that overexpression of *GmNHX6* reduced the damage of NaHCO_3_ treatment on soybean plants.

### Ectopic expression of *GmNHX6* in *Arabidopsis* enhanced the alkaline salt tolerance by maintaining low Na^+^/K^+^ ratios

**Figure 4:**
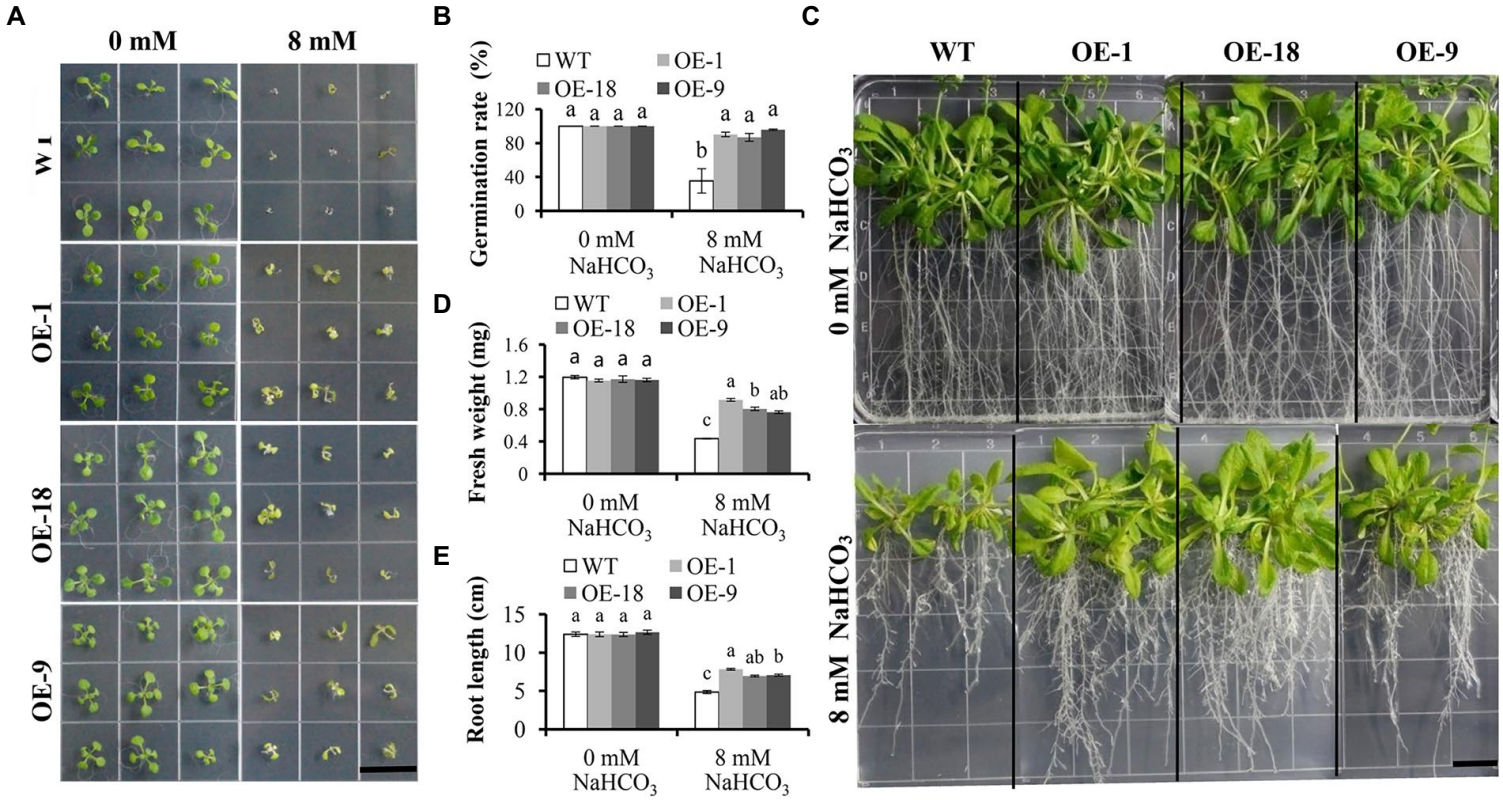
Effect of *GmNHX6* overexpression on *Arabidopsis* tolerance to NaHCO_3_ treatment. **(A)** Seed germination assay of different *Arabidopsis* lines on 1/2 MS medium and 1/2 MS supplied with 8 mM NaHCO_3_. Photographs were taken 7 days after NaHCO_3_ treatment. WT: wild type; OE-1, OE-18, OE-9: *Arabidopsis* lines overexpressing *GmNHX6*. Bar = 1.5 cm. **(B)** Seed germination rates of different *Arabidopsis* lines as shown in A. Data represents the mean ± standard deviation of three biological replications and each repeat contained 36 seeds per line for each treatment (*n* = 36 × 3 = 108). **(C)** Phenotypes of *Arabidopsis* lines subjected to 0 and 8 mM NaHCO_3_ treatment, respectively. Seeds were grown vertically on 1/2 MS agar plates supplemented with 0 or 8 mM NaHCO_3_ for 10 days after seven days of normal growth. Bar = 1.5 cm. **(D,E)** Fresh weight and root length of *Arabidopsis* lines as shown in **(C)**. Data represents the mean ± standard deviation of three biological replications and each repeat contained four plants per line for each treatment (*n* = 12). Bars with the same letter in lowercase above bars indicate no significant differences at the 0.05 level between lines under same treatment according to Duncan’s multiple range tests.

We further investigate the role of *GmNHX6* in response to alkaline salt stress by transgenic *Arabidopsis* plants. *GmNHX6* was overexpressed (OE) using the CaMV 35S promoter in *Arabidopsis*. Seven homozygous transgenic *Arabidopsis* lines containing 35S:*GmNHX6* were obtained at T_3_ generation. Then three lines with higher *GmNHX6* expression levels (OE-1, OE-18, and OE-9) were selected for further analyses (Supplementary Figure 6). The germination rates of *Arabidopsis* lines were compared in the absence or presence of NaHCO_3_, respectively. The results showed that there was no difference in germination rates between the wild type (WT) and *GmNHX6* OE lines under normal condition without NaHCO_3_, but the germination rates of *GmNHX6* OE lines were significantly higher than that of WT *Arabidopsis* under NaHCO_3_ stress (Figures 4A,B). To further test the effect of NaHCO_3_ stress on *Arabidopsis*, we measured the fresh weight and root length of *Arabidopsis*. Under normal condition, no significant difference was observed between *GmNHX6* OE lines and WT (Figures 4C–E). After 10 days of 8 mM NaHCO_3_ treatment, *GmNHX6* OE lines showed better growth than WT (Figure 4C): the average fresh weight and root length of *GmNHX6* OE lines were significantly larger than those of WT *Arabidopsis* (Figures 4D,E). These results suggest that overexpression of *GmNHX6* enhanced the alkaline salt tolerance in *Arabidopsis*.

**Figure 5:**
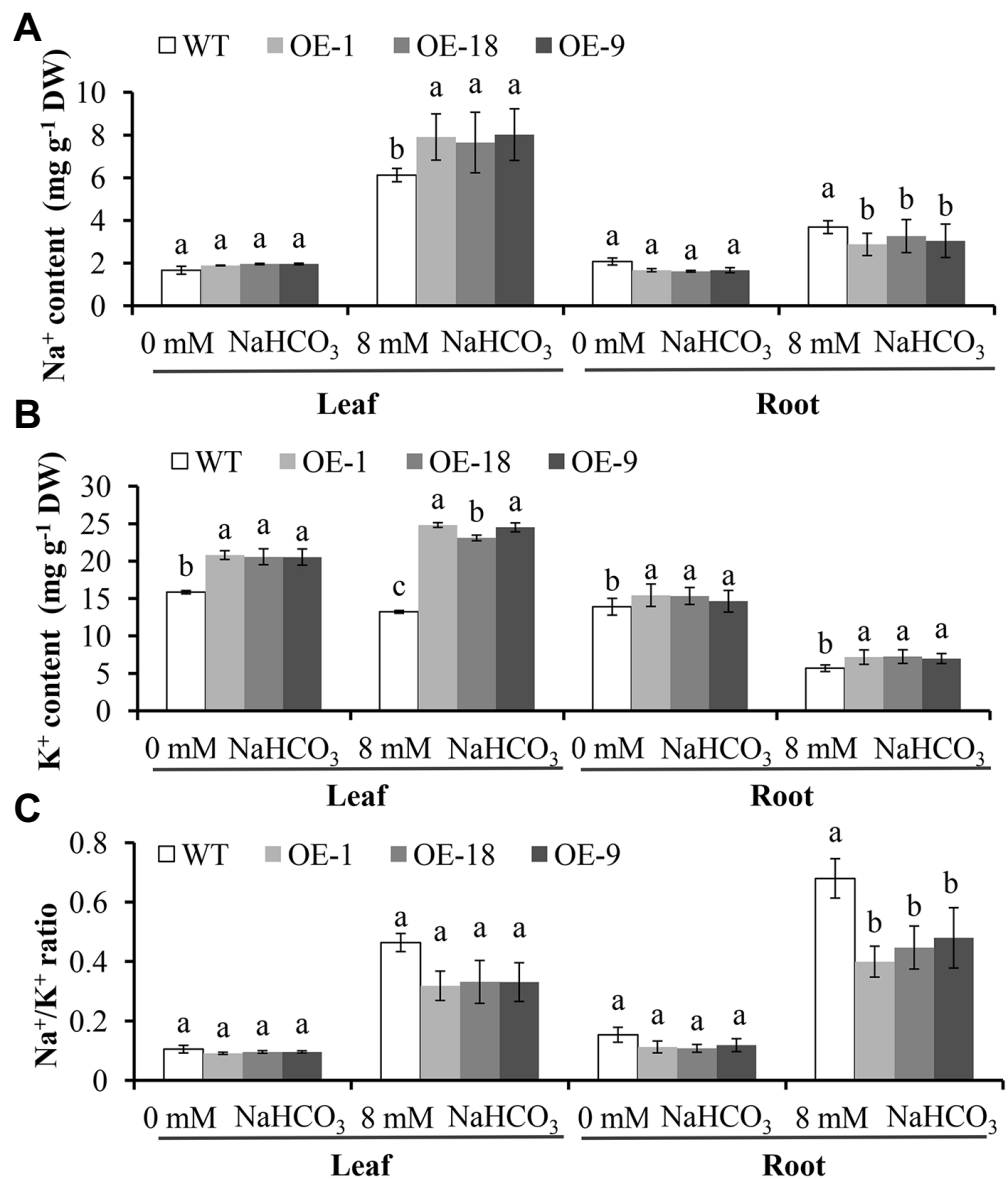
Na^+^ content **(A)**, K^+^ content **(B)**, and Na^+^/K ^+^ ratio **(C)** in the leaves and roots of *Arabidopsis* lines. *Arabidopsis* lines receiving 0 mM or 8 mM NaHCO_3_ treatment for 10 days, respectively. Data represents the mean ± standard deviation of three biological replications and each repeat contained 10 plants per line for each treatment (*n* = 30). Bars with the same letter in lowercase above bars indicate no significant differences at the 0.05 level between lines under same treatment according to Duncan’s multiple range tests.

We also compared the content of Na^+^, K^+^, and the Na^+^/K^+^ ratio in the roots and leaves of *GmNHX6* OE lines with WT *Arabidopsis* under NaHCO_3_ treatment and normal conditions (Figure 5). For Na^+^, under normal conditions, its content in leaf or root was similar between all lines. After NaHCO_3_ treatment, the leaf Na^+^ content in the *GmNHX6* OE lines was significantly higher than that in the WT *Arabidopsis*, but the Na^+^ content in root showed the opposite pattern that OE lines had lower Na^+^ content than WT (Figure 5A). For K^+^, its content in both leaf and root of the *GmNHX6* OE lines was significantly higher than that in the WT before and after NaHCO_3_ treatment (Figure 5B). For Na^+^/K^+^ ratios, there was no significant difference between *GmNHX6* OE lines and WT under normal conditions in both leaves and roots. However, the Na^+^/K^+^ ratio of *GmNHX6* OE lines was lower than that in the WT (Figure 5C). These results suggested that overexpression of *GmNHX6* could improve the alkaline salt tolerance by keeping high K^+^ content and maintaining low Na^+^/K^+^ ratio in plants.

### Natural variation in the promoter region of soybean *NHX6* is associated with alkaline salt tolerance

**Figure 6:**
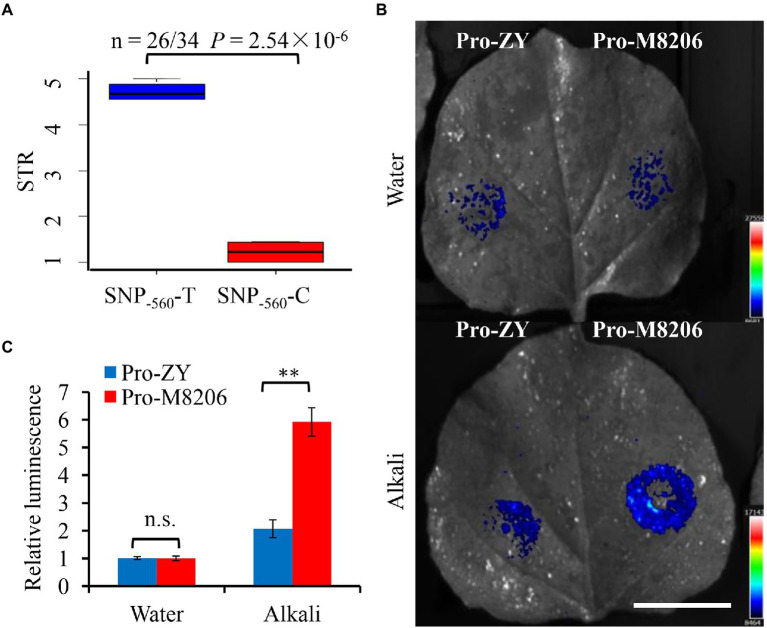
Natural variation and activity of soybean *NHX6* promoter. **(A)** Boxplot of STR for two alleles of SNP_−560_ in *GmNHX6* promoter among 60 soybean accessions. *n* denotes number of accessions. Statistical significance was detected by two-sided Wilcoxon test. The center bold line represents the median; box edges indicate the upper and lower quantiles; whiskers show the 1.5 × interquartile range. STR: sodic (alkaline salt) tolerance rating. **(B,C)** Promoter activities of two types of *GmNHX6* promoters by luciferase (LUC) transient expression assays in tobacco leaves after alkaline salt (100 mM NaHCO_3_) treatment for 16 h. Pro-M8206 contains SNP_−560_-C and Pro-ZY contains SNP_−560_-T. The *LUC* reporter gene was driven by each type of promoter. The photos were taken using an *in vivo* plant imaging system. Bar = 3 cm. Data represents the mean ± standard deviation of four biological replications with two repeats within each replication (*n* = 4 × 2 = 8). Differences were evaluated using the two-tailed Student’s *t*-tests (^**^p < 0.01; n.s., not significant).

We sequenced the coding and promoter regions of soybean *NHX6* from 30 wild soybean (15 alkaline salt tolerant and 15 alkaline salt sensitive soybean accessions) and 30 cultivated soybean (15 alkaline salt tolerant and 15 alkaline salt sensitive), respectively (Supplementary Table 1; Supplementary Figure 7). The results showed that only the *NHX6* promoter region had sequence variation among 60 soybean accessions. A total of 30 SNPs were found in the 2-kb promoter region of soybean *NHX6* (Supplementary Figure 8). Then, a regional association study was performed using the 30 SNPs in the 60 soybean accessions with extreme sodic tolerance rating (STR). Only one SNP (SNP_−560_, 560 bp upstream of the start codon) showed significant association with STR by mixed model in EMMAX (Supplementary Figure 9; Supplementary Table 4). Among the 60 soybean accessions, the majority (25 out of 30, 83%) of alkaline salt tolerant accessions had a base “C” at SNP_−560_ upstream of *GmNHX6*, while 70% (21 out of 30) alkaline salt sensitive accessions had a base “T” at SNP_−560_ (Supplementary Figure 8). There was a significant difference in alkaline salt tolerance between the two groups of SNP_−560_-C and SNP_−560_-T in 60 soybean accessions, as well as in wild soybean accessions and cultivated soybean accessions (Figure 6A; Supplementary Figure 10). In order to further compare the two types of promoters containing SNP_−560_-C and SNP_−560_-T, the representative promoters were cloned from two soybean varieties, M8206 and ZY, and designated as Pro-M8206 (containing SNP_−560_-C) and Pro-ZY (containing SNP_−560_-T), respectively. The promoter-LUC transient expression assays in tobacco leaves revealed that the activity of Pro-M was significantly stronger than that of Pro-ZY under alkali stress but not control condition (Figures 6B,C), suggesting that Pro-M is more responsive to alkali stress than Pro-ZY, therefore, leading to higher alkali induced expression level of *GmNHX6* in the alkaline salt tolerant soybean accessions than sensitive accessions, thus resulting in enhanced alkaline salt tolerance.

## Discussion

Soil salinization and alkalization can reduce soil osmotic potential, and cause ion imbalance, disrupt physiological processes, inhibit growth and development of plants, leading to a serious decline in its yield and quality, and even the death of plants (Zhu, 2001; Yang et al., 2011). Soil salinization and alkalization frequently co-occur, but in the saline-alkaline land in mainland China, the soil salinization caused by alkali salts such as NaHCO_3_ and Na_2_CO_3_ is more serious than that caused by neutral salts such as NaCl and Na_2_SO_4_ (Yang et al., 2008, 2011). In general, the stress factors of neutral salts are mainly the ion stress of Na^+^ and osmotic stress of low water potential caused by high salt concentration, but for the alkaline salts, there is an added factor of high pH (Shi et al., 1998). The alkaline soil causes damage to plants not only through salt stress but also through high pH (Shi and Wang, 2005). Therefore, the deleterious effect of high pH stress or salinity alone is significantly less than that of the combined stress of high pH and salinity (Li et al., 2010). There have been lots of progress on plant tolerance to salt stress, while only few reports on alkali salt tolerance. Here, we investigated the role of soybean *NHX6* gene in plant tolerance to alkaline salt (NaHCO_3_), which would broaden our knowledge on alkali salt tolerance.

In plants, sodium hydrogen exchangers allow cation/H^+^ such as Na^+^/H^+^ to transmembrane transport, to regulate pH and maintain ion homeostasis to resist abiotic stress (Rodríguez-Rosales et al., 2009; Bassil et al., 2012). However, there is no report about its role in soybean tolerance to alkali stress. In this study, a sodium/hydrogen exchanger gene from the soybean, *GmNHX6*, was found to be involved in the alkaline salt tolerance of soybean. The expression of *GmNHX6* was induced by NaHCO_3_ treatment and more upregulated in alkali salt tolerant soybean (Figure 2), suggesting it is involved in plant responses to alkaline stress. Transgenic soybean composite plants and *Arabidopsis* plants have been previously used to study gene functions in saline-alkaline tolerance (Al-Harrasi et al., 2020; Ketehouli et al., 2021). *GmPKS4* overexpressing soybean composite plants and transgenic *Arabidopsis* plants had increased proline content as well as high antioxidant enzyme activities under salt and salt-alkali stress treatments, compared to the empty-vector-transformed or wild-type ones (Ketehouli et al., 2021). Then we employed transgenic soybean composite plants and *Arabidopsis* to investigate the role of *GmNHX6* in alkaline salt tolerance (Figures 3–5; Supplementary Figure 5). We found that overexpression of *GmNHX6* enhanced the alkaline salt tolerance of soybean and *Arabidopsis*.

**Figure 7:**
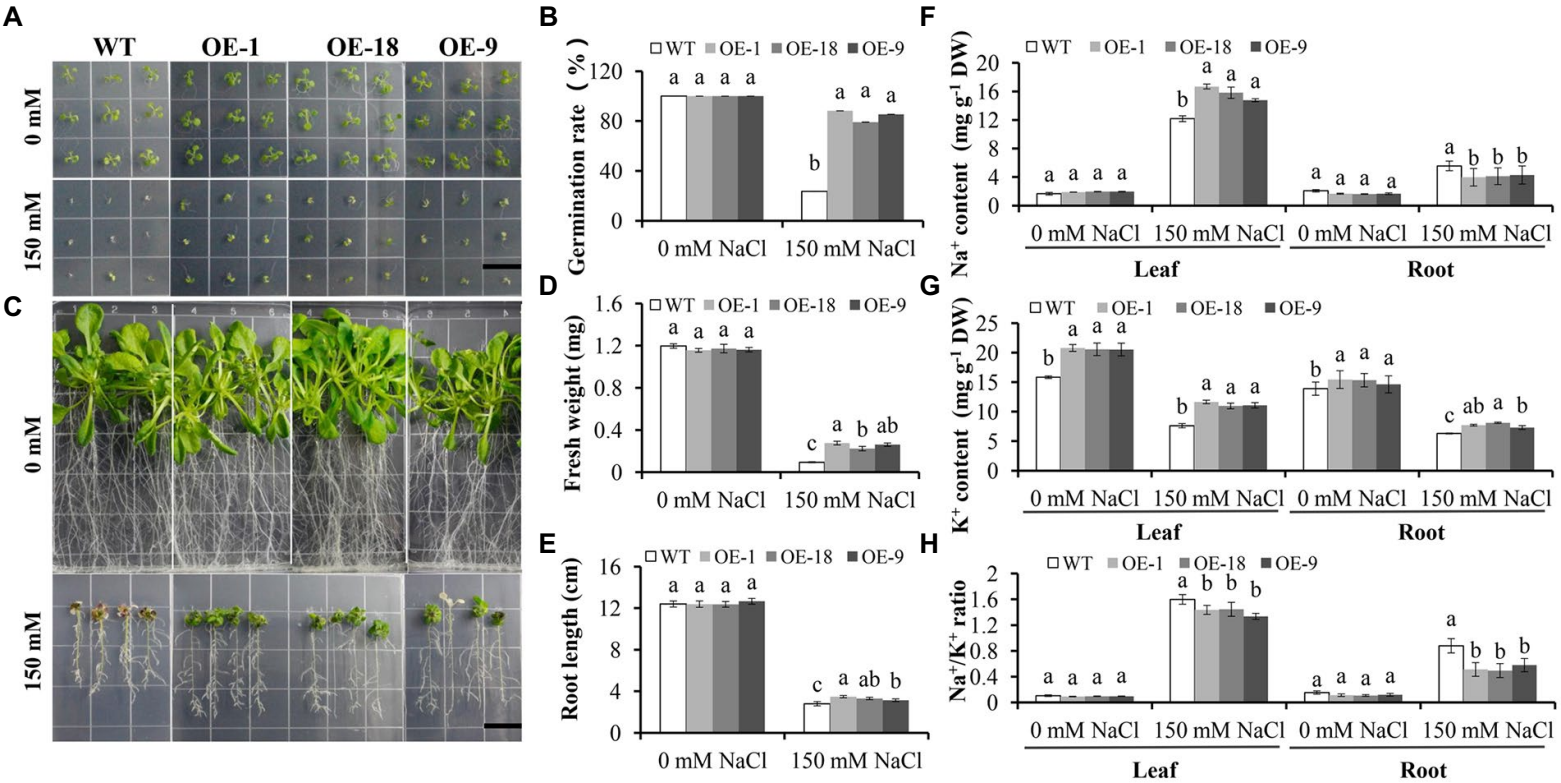
The effect of *GmNHX6* overexpression on the salt tolerance of *Arabidopsis*. **(A)** Seed germination assay of different *Arabidopsis* lines on 1/2 MS medium supplied with 0 or 150 mM NaCl. Photographs were taken 7 days after treatment. WT: wild type; OE-1, OE-18, OE-9: *Arabidopsis* lines overexpressing *GmNHX6*. Bar = 1.5 cm. **(B)** Seed germination rates of different *Arabidopsis* lines as shown in A. Data represents the mean ± standard deviation of three biological replications and each repeat contained 36 seeds per line for each treatment (*n* = 36 × 3 = 108). **(C)** *Arabidopsis* seeds were grown vertically on 1/2 MS agar plates supplemented with 0 or 150 mM NaCl treatment for 10 days after 7 days of normal growth, respectively. Bar = 1.5 cm. **(D,E)** The fresh weight and root length of *Arabidopsis* lines shown in **(C)**. Data represents the mean ± standard deviation of three biological replications and each repeat contained four plants per line for each treatment (*n* = 12). **(F)** Na^+^ content, **(G)** K ^+^ content, **(H)** Na^+^/K ^+^ ratio in the leaves and roots of *Arabidopsis* lines shown in **(C)**. For **(F–H)**, data represents the mean ± standard deviation of three biological replications and each replication has four repeats (*n* = 12), and 10 plants were pooled together for each sample. Bars with the same letter in lowercases above bars indicate no significant differences at the 0.05 level between lines under each condition according to Duncan’s multiple range test.

Since *NHX* genes have been shown to play important roles in salt tolerance in a variety of plant species (Kobayashi et al., 2012; Al-Harrasi et al., 2020; Long et al., 2020; Sun et al., 2021), we further analyzed the function of *GmNHX6* in salt tolerance. The germination rates of *GmNHX6* OE lines were significantly higher than that of WT *Arabidopsis* under NaCl stress (Figures 7A,B). At seedling stage, overexpression of *GmNHX6* in *Arabidopsis* increased the average fresh weight and root length under salt stress (Figures 7C–E). We also compared the contents of Na^+^ and K^+^ in the roots and leaves of *Arabidopsis* (Figures 7F–H). Under normal conditions, there is no significant difference in the content of Na^+^ content between WT and OE lines. However, under NaCl treatment, the Na^+^ content in the leaves of the *GmNHX6* OE lines was significantly higher than that in the WT, but in the root Na^+^ content was just the opposite (Figure 7F). The K ^+^ content of *GmNHX6* OE lines was significantly higher than that in the WT *Arabidopsis* in both leaves and roots (Figure 7G). The Na^+^/K^+^ ratios of the *GmNHX6* OE lines were significantly lower than that in the WT *Arabidopsis* under NaCl treatment (Figure 7H). These results suggest that overexpression of *GmNHX6* enhanced salt tolerance by maintaining Na^+^ and K^+^ homeostasis.

Different from salt stress, alkali stress caused by NaHCO_3_ and Na_2_CO_3_ leads to higher pH (Fang et al., 2021). According to the previous studies, the NHXs are important regulators of cellular pH and ion homeostasis (Rodríguez-Rosales et al., 2009; Bassil et al., 2012). NHX-type antiporters utilize the H^+^ electrochemical gradient to facilitate the exchange of H^+^ for cations such as Na^+^ or K^+^, thereby maintaining both pH and ion homeostasis (Sze and Chanroj, 2018). Studies also showed that plants can secrete large amounts of organic acids under alkali stress, which can play a buffer role and maintain intracellular pH stability (Fang et al., 2021). However, further experiments are needed to investigate the role of *GmNHX6* in adjusting pH to alleviate alkaline salt stress.

K^+^ is an essential macronutrient for plant growth and the most abundant inorganic cation in plant cells. During plant growth and development, K^+^ is involved in the activation of more than 60 enzyme systems, photosynthesis, carbohydrate metabolism and protein synthesis, what’s more, K^+^ is a key modulator for cell homeostasis (Zhang et al., 2019a; Kumar et al., 2020). Na^+^ toxicity is one of the main harmful factors of saline-alkali stress (Apse and Blumwald, 2007). In root cells, Na^+^ is compartmentalized into vacuoles, radial transported to the stele cells, and loaded into the xylem, thus establishing the homeostatic control of Na^+^ in the cytosol (Apse and Blumwald, 2007). Plants can maintain high K^+^ content by increasing K^+^ absorption and reducing K^+^ loss (Zhu, 2003). Under saline-alkali stress, plant cells absorb a large amount of Na^+^, resulting in ion toxicity, which not only inhibits photosynthesis, but also inhibits the absorption of essential element such as K^+^ by plants, when Na^+^/K^+^ is too high, resulting in the change of ion homeostasis and metabolic disorder (Zhu, 2003). Therefore, maintaining a relatively high K^+^ content and low Na^+^/K^+^ ratio in plants under salt-alkali stress is a manifestation of salt-alkali tolerance. The NHXs play important roles in maintaining Na^+^ and K^+^ homeostasis have been well documented (Wang et al., 2015; Sun et al., 2021). Previous studies have shown that NHXs can compartmentalize Na^+^ into vacuoles under salt stress, thus maintaining the intracellular ion balance (Feng et al., 2021). Other studies found that vacuolar NHXs can simultaneously catalyze the exchange of Na^+^/H^+^ and K^+^/H^+^ to maintain intracellular ion balance (Sze and Chanroj, 2018). Under salt stress, NHX transporter, such as NHX1, transport sodium ions to and sequester them in the vacuole by vesicle transport, and release K^+^ from vacuolar into the cytoplasm (Barragán et al., 2012). In *Arabidopsis, AtNHX5* and *AtNHX6* are critical to K^+^ homeostasis in *Arabidopsis* (Wang et al., 2015). *GmNHX5* positively regulates salt tolerance by maintaining higher K^+^/Na^+^ ratio in soybean (Sun et al., 2021). In this study, we found that overexpression of *GmNHX6* enhanced *Arabidopsis* tolerance to both alkaline salt (NaHCO_3_) and salt (NaCl) stress, by maintaining higher K^+^ content and low Na^+^/K^+^ ratios.

We also obtained a homozygous mutant *nhx6* (stock, *SALK-100042C*), a T-DNA insertion *Arabidopsis* mutant of *AtNHX6* (Supplementary Figure 11). However, *nhx6* mutant did not show alkaline salt tolerance or salt tolerance compared with the WT *Arabidopsis* (Supplementary Figure 12), which is consistent with the previous study by Bassil et al. (2011a) that the phenotype of *nhx5* or *nhx6* single-knockout line was not different from the WT, while the double mutant *nhx5 nhx6* has reduced growth, smaller and fewer cells, and increased sensitivity to salinity. Through the EnsemblPlants database,^11^ we identified a total of 66 genes in the soybean *NHX* gene family, which might be functionally redundant in soybean tolerance to salt-alkali stress.

Soybean germplasm provides a wide range of saline-alkaline tolerance. To explore the natural variation in soybean *NHX6* gene, we analyzed the sequence polymorphism of the 2-kb promoter region (Supplementary Figure 13) of *NHX6* in 60 soybean accessions, including *G. max* and *G. soja*. Only one SNP (SNP_−560_) out of a total of 30 SNPs showed significant association with alkaline salt tolerance (STR) by mixed model in EMMAX (Supplementary Table 4). We analyzed the *cis*-elements of the 2-kb promoter region (Supplementary Figure 13) of *GmNHX6* in two soybean accessions, M8206 and ZY. We found that in Pro-M8206 (containing SNP_−560_-C), the sequence forms four cis-acting elements around SNP_−560_, including Skn-1_motif (GTCAT, −562 to −558 bp), TCATTT element (−561 to −556 bp), TTGTCA motif (−564 to −559 bp), and TGTCAT motif (−563 to −559 bp). Skn-1_motif is responsive to abiotic stress and could be used in plant genetic engineering research on abiotic stress tolerance (De Silva et al., 2017; Zhang et al., 2018). TTGTCA element is involved in light induction (Mazouni et al., 2003). Previous studies showed that TCATTT element and TGTCAT element are the binding sites of downstream key functional genes. TCATTT-containing element acted as an enhancer (Perrotti et al., 1996), and was essential for inducible expression of the *IL-5* gene (Miao et al., 2006). Tunicamycin (Tm)-activated sXBP1 bound to the TGTCAT element and suppressed *XRCC2* expression to prevent tumor proliferation *in vivo* (Zhao et al., 2021). Interesting, promoter-LUC transient expression assays in tobacco leaves revealed that the promoter of *GmNHX6* from the alkaline salt tolerant soybean variety, Pro-M8206, had a significantly stronger activity than Pro-ZY (from alkaline salt sensitive variety), under alkali stress (Figures 6B,C), suggesting that Pro-M8206 is more responsive to alkali stress than Pro-ZY. Therefore, it is likely that the SNP_−560_ might affect the promoter activity under alkali stress by interrupting the relevant *cis*-acting elements mentioned above. How these *cis*-acting elements in the promoter of *GmNHX6* regulate gene expression in response to alkali stress needs further study.

## Conclusion

In summary, *GmNHX6* encodes a sodium/hydrogen exchanger. The expression of *GmNHX6* was induced by NaHCO_3_ stress, and greater increase in its transcript abundance was observed in alkaline salt tolerant than in alkaline salt sensitive soybean variety. Overexpression of *GmNHX6* enhanced the alkaline-salt tolerance of soybean composite plants and *Arabidopsis*. The *GmNHX6* overexpressing *Arabidopsis* lines had higher K^+^ content and lower Na^+^/K^+^ ratio than the wild-type plants under NaHCO_3_ stress. Furthermore, a single nucleotide polymorphism in the promoter region of *NHX6* is associated with the alkali tolerance in soybean. These findings would help to further understand the role of *NHX6* and its regulatory mechanism in soybean tolerance to alkaline-salt stress.

## Data availability statement

The datasets presented in this study can be found in online repositories. The names of the repository/repositories and accession number(s) can be found in the article/Supplementary material.

## Author contributions

TJ and YL conceived and designed the experiments, interpreted the results, and wrote and revised the manuscript. TJ, JA, HX, JC, LP, RZ, and NW performed the experiments. TJ analyzed the data and generated the pictures. JG and YL contributed reagents and materials. All authors contributed to the article and approved the submitted version.

## Funding

This work was supported by the National Key Research and Development Program of China (2021YFF1001204) and the Core Technology Development for Breeding Program of Jiangsu Province (JBGS-2021-014).

## Conflict of interest

The authors declare that the research was conducted in the absence of any commercial or financial relationships that could be construed as a potential conflict of interest.

## Publisher’s note

All claims expressed in this article are solely those of the authors and do not necessarily represent those of their affiliated organizations, or those of the publisher, the editors and the reviewers. Any product that may be evaluated in this article, or claim that may be made by its manufacturer, is not guaranteed or endorsed by the publisher.

## Footnotes

^1^ http://soybase.org

^2^ http://www.premierbiosoft.com/primerdesign/

^3^ https://phytozome.jgi.doe.gov/pz/portal.html

^4^ https://www.ebi.ac.uk/Tools/msa/clustalw2/

^5^ http://bioinformatics.psb.ugent.be/webtools/plantcare/html/

^6^ https://wlab.ethz.ch/protter/start/

^7^ http://www.arabidopsis.org

^8^ https://imagej.nih.gov/ij/

^11^ http://plants.ensembl.org/biomart/martview/
