# Supplementary material for: A soybean sodium/hydrogen exchanger GmNHX6 confers plant alkaline salt tolerance by regulating Na+/K+ homeostasis
Source: Front Plant Sci. 2022 Sep 20;13:938635. doi: 10.3389/fpls.2022.938635 (PMC9531905; doi:10.3389/fpls.2022.938635)
Supplement: Supplementary file 1 [file Data_Sheet_1.PDF]

Supplementary Material

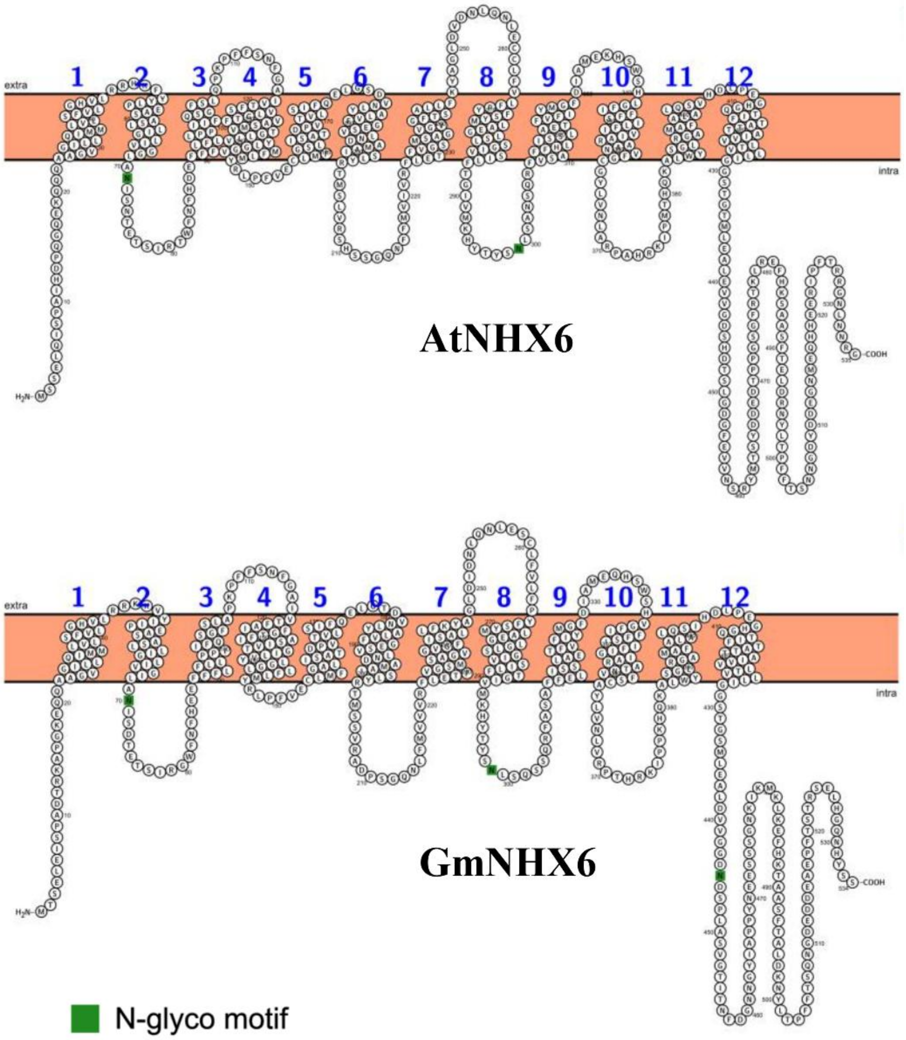

Supplementary Figure 1. The putative topology of AtNHX6 and GmNHX6 using Protter database. The topology of GmNHX6 of soybean shows 12 transmembrane domains, similar with that of AtNHX6 of *Arabidopsis*.

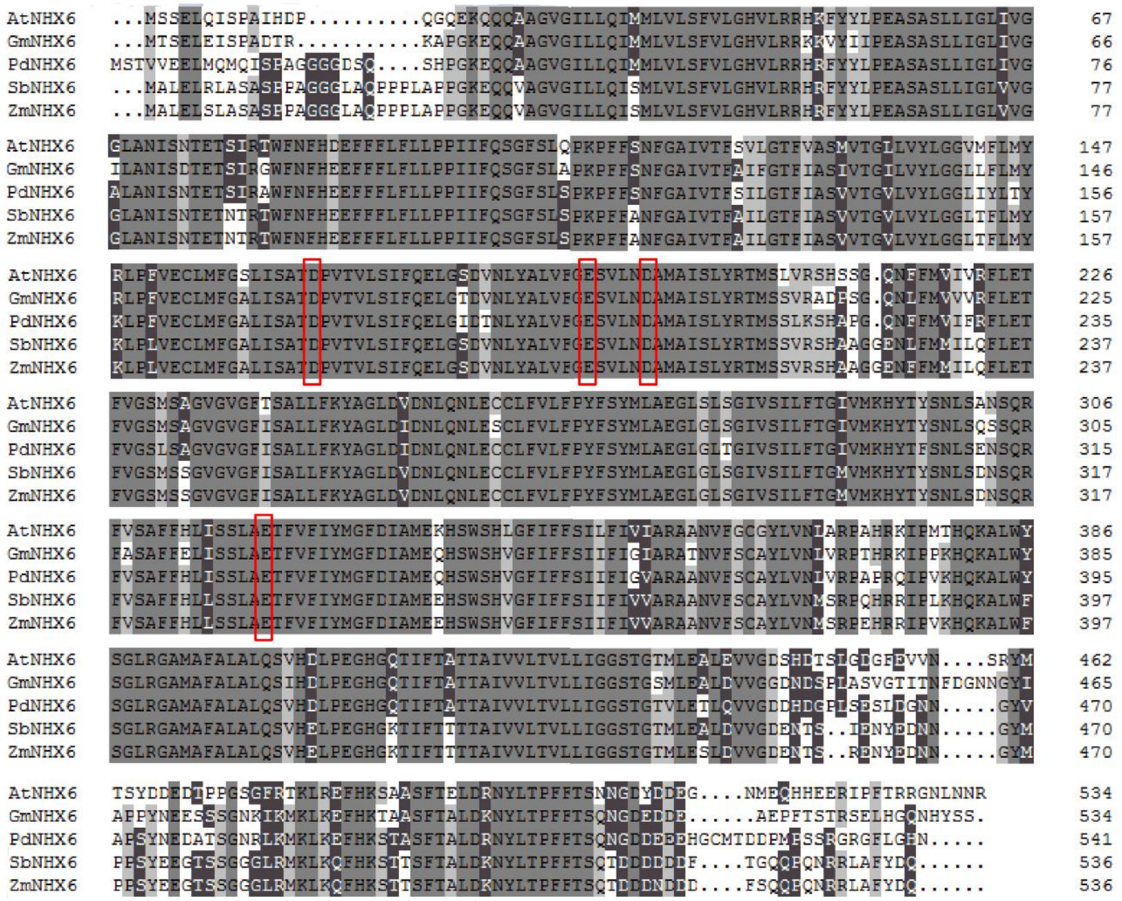

Supplementary Figure 2. Multiple sequence alignment of the deduced amino acid sequence of GmNHX6 and four NHX6 sequences from different plant species. Red boxes are the conserved acidic residues of NHX6. Four other NHX6 sequences include AtNHX6 (NP_178079.2) from *Arabidopsis thaliana*, PdNHX6 (XP_008811204.1) from *Phoenix dactylifera*, SbNHX6 (XP_021308686.1) from *Sorghum bicolor*, ZmNHX6 (ACG45634.1) from *Zea mays*.

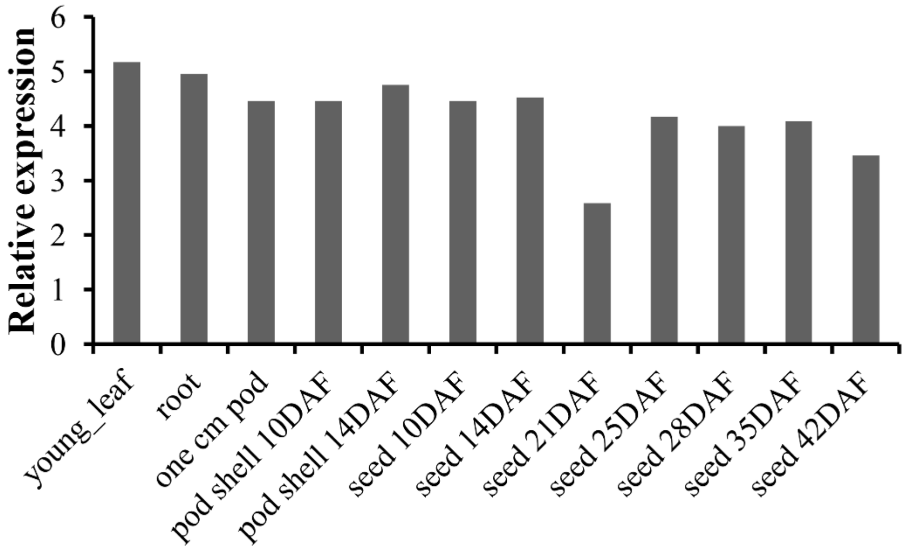

Supplementary Figure 3. Tissue expression patterns of *GmNHX6* in soybean. The raw reads from RNA-seq data of *Glycine max* (www.soybase.org) were transformed to $\log_2$ (FPKM + 1).

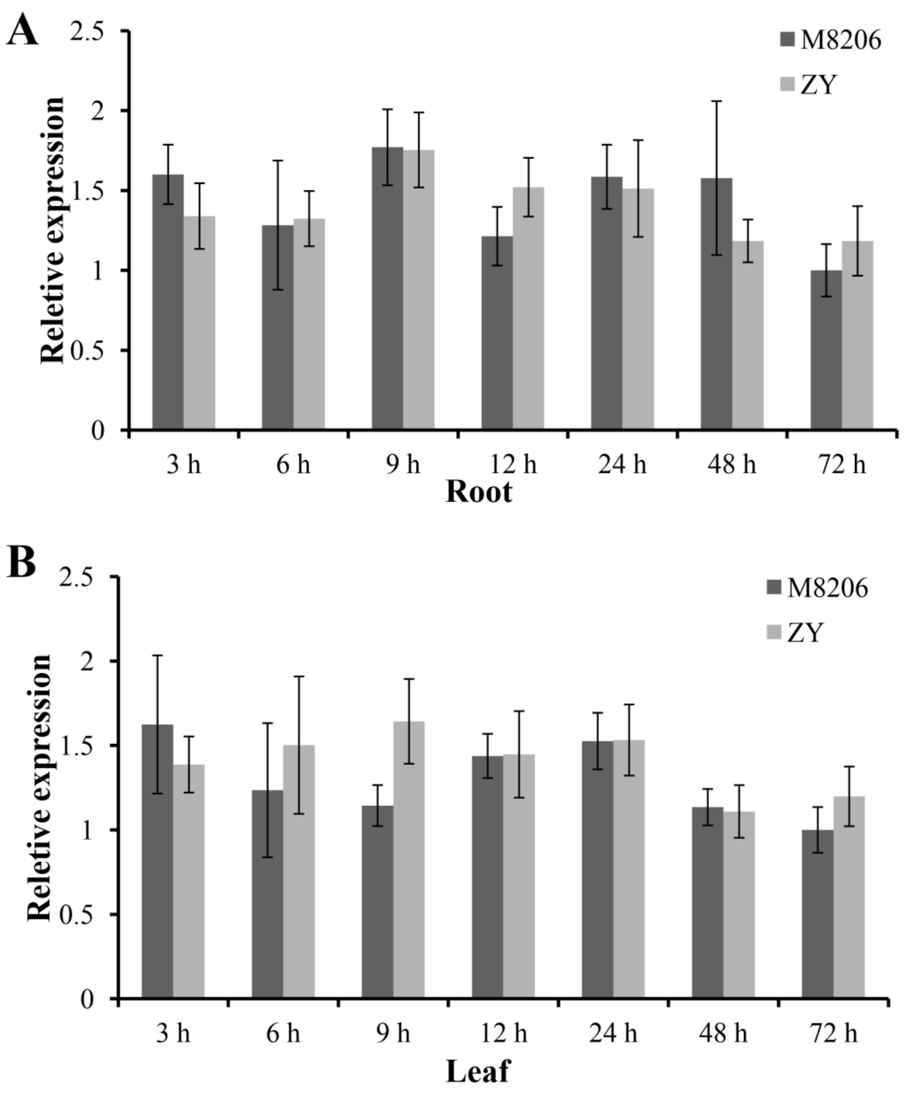

Supplementary Figure 4. Relative expression of *GmNHX6* in two soybean varieties under normal condition (0 mM $NaHCO_3$). The relative expression of *GmNHX6* in soybean root tips (0 ~ 2 mm) was quantified by qRT-PCR, using *GmUKN1* as the reference gene and M8206 under 0 mM $NaHCO_3$ at 72 h (with lowest expression level of *GmNHX6*) as the control (relative expression = 1). Data represents the mean ± standard deviation of three replicates. There was no difference between two soybean varieties by the two-tailed Student's *t* tests.

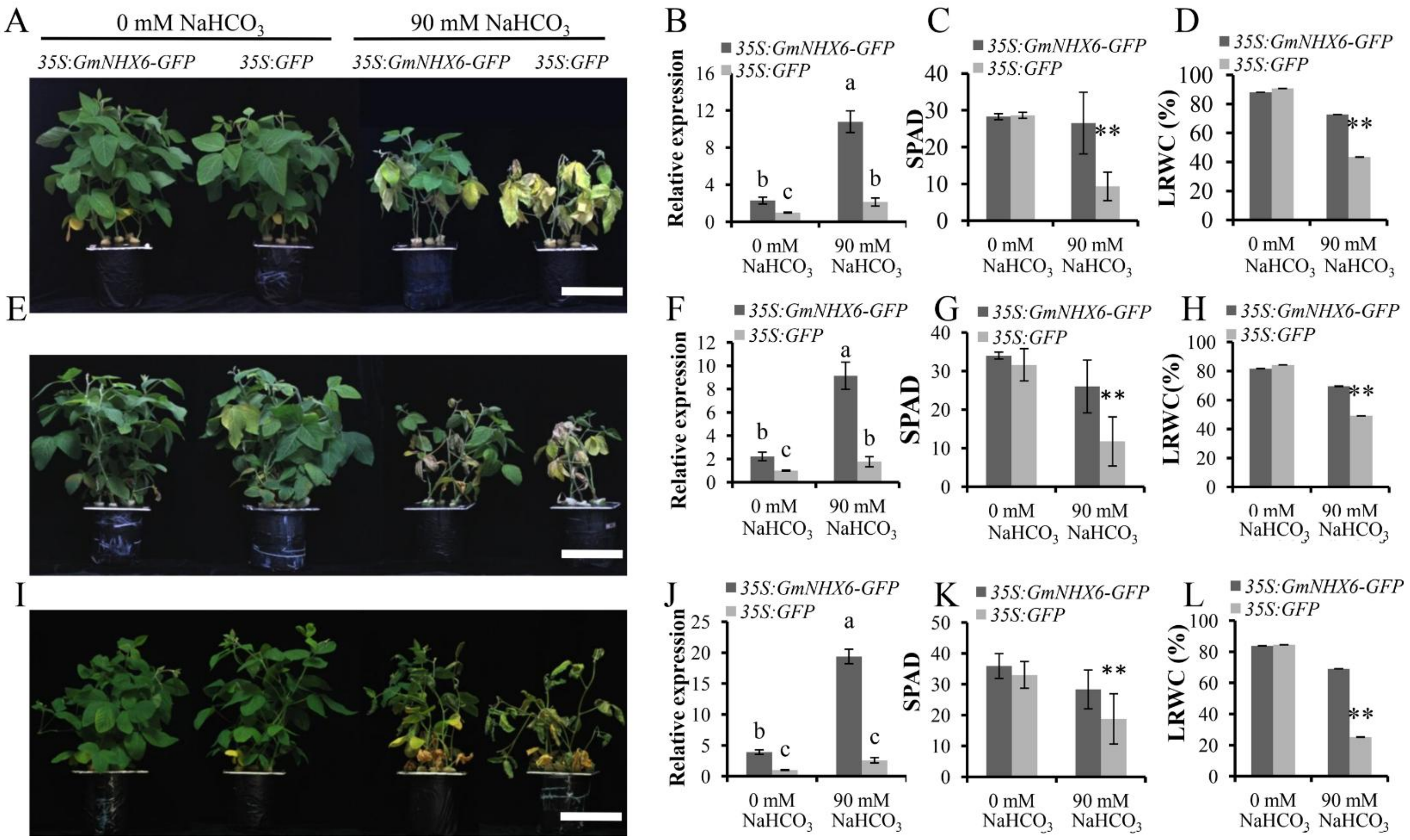

Supplementary Figure 5. Three biological replications for salt tolerance analyses of transgenic soybean composite plants. The phenotypes (**A, E, I**), relative expression (**B, F, J**), SPAD values for chlorophyll content (**C, G, K**) and leaf relative water content (**D, H, L**) of transgenic soybean composite plants under control (0 mM $NaHCO_3$) or alkali stress (90 mM $NaHCO_3$) were recorded 7 d after treatment. Bar, 12 cm. Soybean variety of "TianLong1" was used. Data represents the mean ± standard deviation of technical repeats and each repeat has at least five independent plants for each genotype (n = 5). Same letters above bars indicate no significant differences according to Duncan's multiple range test at 0.05 level. $^{**}P < 0.01$ by two-tailed Student's t tests.

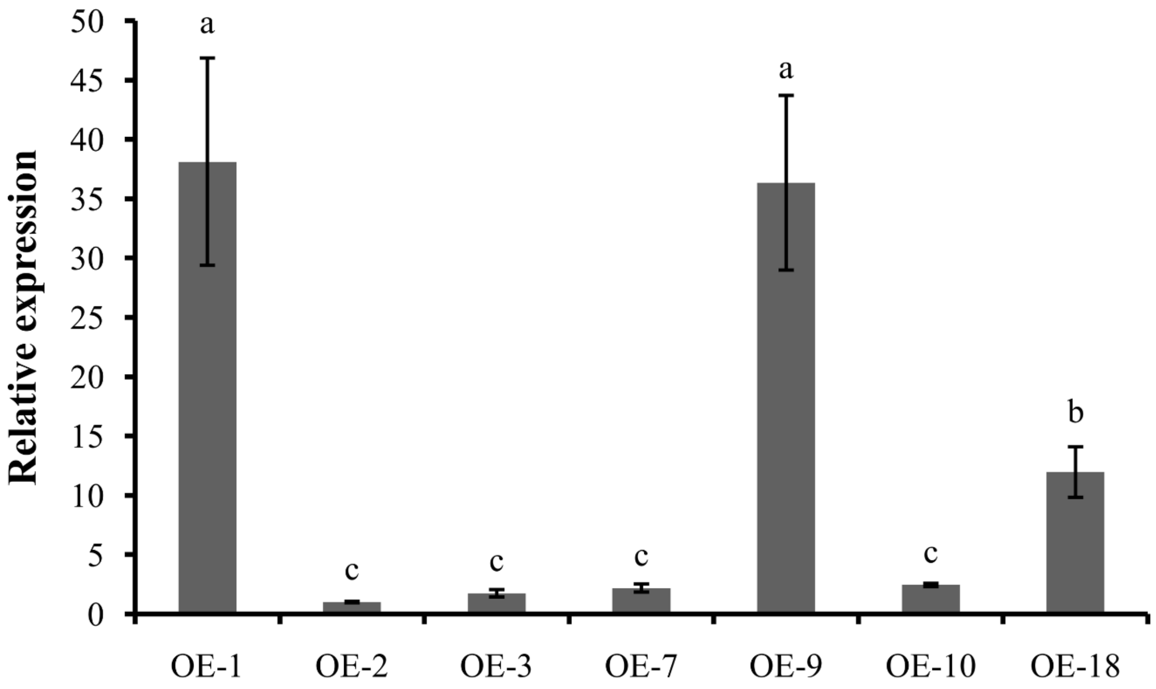

Supplementary Figure 6. The relative expression level of *GmNHX6* in transgenic *Arabidopsis* lines. The relative expression of *GmNHX6* in transgenic *Arabidopsis* lines was quantified by qRT-PCR, using *AtACTIN7* as the reference gene and OE-2 line (with lowest expression level of *GmNHX6*) as the control (relative expression = 1). Data represents the mean ± standard deviation of three replicates.

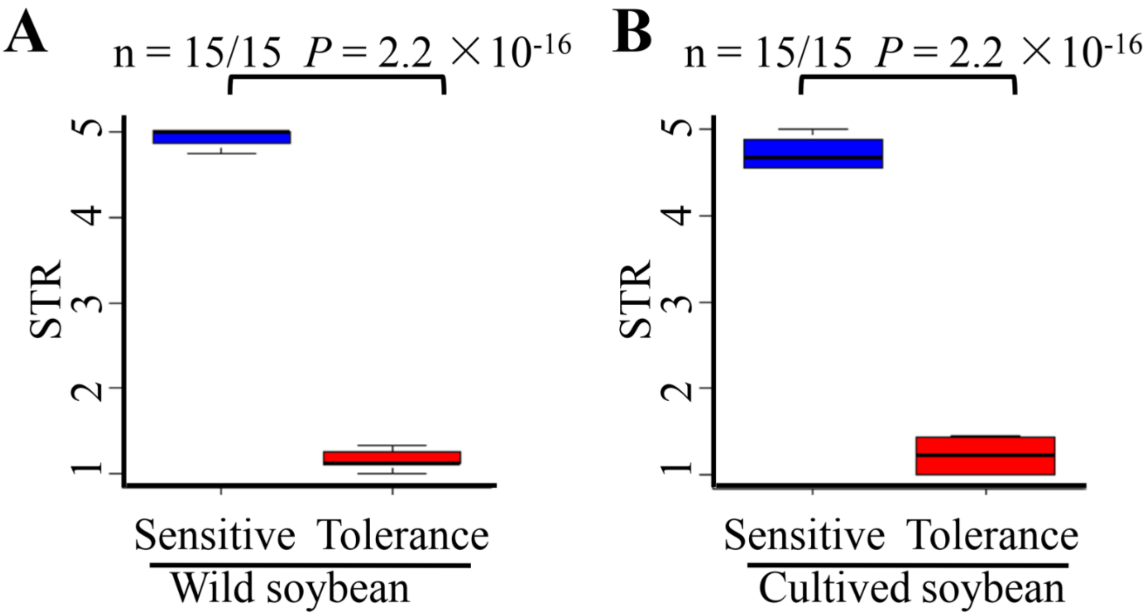

Supplementary Figure 7. Boxplots of alkaline salt tolerance in 30 wild soybean accessions and 30 cultivated soybean accessions. STR: Sodic (alkaline salt) Tolerance Rating.

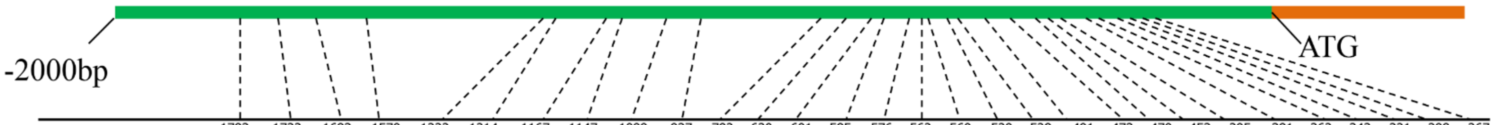

			-1783	-1733	-1683	-1570	-1222	-1214	-1167	-1147	-1099	-927	-782	-630	-601	-585	-576	-562	-560	-529	-528	-491	-473	-470	-453	-395	-381	-362	-343	-321	-298	-267
Wild Soybean	Tolerance	LY01-10	C	A	C	G	T	A	A	G	C	A	T	G	T	A	G	G	C	G	G	T	G	G	G	A	C	G	G	T	T	C
		LY15-07	C	A	C	G	T	A	A	G	C	A	T	G	T	A	G	G	C	G	G	T	G	G	G	A	C	G	G	T	T	C
		LY04-02	C	G	C	G	A	A	T	A	G	T	T	G	T	A	G	G	C	G	G	T	G	G	C	A	C	A	A	T	C	T
		LY04-04	C	A	C	G	T	A	A	G	C	A	T	G	T	A	G	G	C	G	A	T	G	G	G	A	C	G	G	T	T	C
		LY05-06	C	G	C	G	A	A	T	A	G	T	T	G	T	A	G	G	C	G	G	T	G	G	C	A	C	A	A	T	C	T
		LY12-01	C	A	C	G	T	A	T	G	C	A	T	G	T	A	G	G	C	G	G	T	G	G	G	A	C	G	G	T	T	C
		LY14-03	C	G	C	G	A	A	T	A	G	T	T	G	T	A	G	G	C	G	G	T	A	G	C	A	C	A	A	T	C	T
		LY14-09	C	A	C	G	T	A	A	G	C	A	T	G	T	A	G	G	C	G	G	T	G	G	G	A	C	G	G	T	T	C
		LY16-06	C	A	C	G	T	A	A	G	C	A	T	G	T	A	G	G	C	G	A	T	G	G	G	A	C	G	G	T	T	C
		LY01-07	C	G	C	G	A	A	T	A	G	T	T	G	T	A	G	G	C	G	G	T	G	G	C	A	C	A	A	T	C	T
		LY04-01	C	G	C	G	A	A	T	A	G	T	T	G	T	A	G	G	C	G	G	T	G	G	C	A	C	A	A	T	C	C
		LY04-03	C	G	C	G	A	A	T	A	G	T	T	G	T	A	G	G	C	G	G	T	G	G	C	A	C	G	A	T	C	T
		LY07-06	C	G	C	G	A	A	T	A	G	T	T	G	T	A	G	G	C	G	G	T	G	G	C	A	C	G	A	T	C	T
		LY15-01	C	G	C	G	A	A	T	A	G	T	T	G	T	A	G	G	C	G	G	T	G	G	C	A	C	A	A	T	C	T
		LY04-06	C	G	T	T	T	T	T	G	C	T	T	G	T	A	G	G	T	G	G	T	G	G	G	A	C	A	A	A	C	C
	Sensitive	LY06-05	C	G	C	G	A	A	T	A	G	T	G	G	T	A	G	G	T	G	G	T	G	G	G	A	C	A	A	T	C	C
		LY07-03	C	G	T	G	T	T	T	A	G	A	G	G	C	G	A	A	T	C	A	C	A	A	G	G	T	A	A	T	C	C
		LY09-09	C	G	T	G	T	A	T	G	C	A	G	G	C	G	A	A	T	C	A	C	A	A	G	G	T	G	A	T	C	C
		LY10-04	C	G	T	T	T	T	T	A	G	T	T	G	T	A	G	G	T	G	G	T	G	G	C	A	C	A	A	A	C	C
		LY16-10	C	G	C	G	T	A	T	G	C	A	G	G	C	G	A	A	T	C	A	C	A	A	G	G	T	A	G	T	C	C
		LY03-05	C	G	C	G	A	A	T	A	G	T	T	G	T	A	G	G	C	G	G	T	G	G	C	A	C	G	A	T	C	T
		LY05-10	C	G	C	G	A	A	T	A	G	T	T	G	T	A	G	G	T	G	G	T	G	G	C	A	C	A	A	T	C	T
		LY10-07	T	G	C	G	A	A	T	A	G	T	T	G	T	A	G	G	T	G	G	T	G	G	G	A	C	G	G	T	C	C
		LY13-09	C	G	C	G	A	A	T	A	G	A	G	G	T	A	G	G	C	G	G	T	G	G	G	A	C	G	G	T	C	C
		LY15-09	T	G	T	T	T	T	T	G	C	T	T	G	T	A	G	G	T	G	G	T	G	G	G	A	C	G	G	A	C	C
		LY16-01	C	G	T	G	T	A	T	G	C	A	G	G	C	G	A	A	C	C	A	C	A	A	G	G	T	A	A	T	C	C
		LY19-01	C	G	T	T	T	A	T	G	C	T	T	G	T	A	G	G	C	G	G	T	G	G	G	A	C	A	A	A	C	C
		LY21-03	T	G	T	T	T	T	T	G	C	T	T	G	T	A	G	G	T	G	G	T	G	G	G	A	C	G	G	A	C	C
		LY10-01	T	G	C	G	A	A	T	A	G	T	T	G	T	A	G	G	T	G	G	T	G	G	C	A	C	A	G	T	C	T
		LY21-01	C	A	C	G	T	A	A	G	C	A	T	G	T	A	G	G	C	G	A	T	G	G	G	A	C	A	A	T	T	C
Cultivated Soybean	Tolerance	Meng8206	C	G	C	G	A	A	T	A	G	T	T	G	T	A	G	G	C	G	G	T	G	G	G	A	C	A	A	T	C	C
		Wuheqihuangdou	C	G	C	G	A	A	T	A	G	T	T	G	T	A	G	G	C	G	G	T	G	G	C	A	C	A	A	T	C	T
		Williams82	T	G	T	T	T	T	T	G	C	T	T	G	T	A	G	G	T	G	G	T	G	G	G	A	C	G	G	A	C	C
		Zhongdou8	C	G	C	G	A	A	T	A	G	T	T	G	T	A	G	G	C	G	G	T	G	G	C	A	C	A	A	T	C	T
		Jindou6	C	G	C	G	A	A	T	A	G	T	T	G	T	A	G	G	C	G	G	T	G	G	C	A	C	G	A	T	C	T
		Erwangdou	C	G	T	T	T	T	T	G	C	T	T	G	T	A	G	G	T	G	G	T	G	G	G	A	C	G	A	A	C	C
		Yudou22	C	G	T	T	T	T	T	G	C	A	G	G	C	G	A	A	C	C	A	C	A	A	G	G	T	G	A	A	C	C
		Pingbuxiaohuangdou	C	G	C	G	A	A	T	A	G	T	T	G	T	A	G	G	C	G	G	T	G	G	C	A	C	G	A	T	C	T
		Gongdou2	C	G	T	T	T	T	T	G	C	T	T	G	T	A	G	G	C	G	G	T	G	G	G	A	C	G	A	A	C	C
		Xudou8hao	C	G	T	T	T	T	T	G	C	T	T	G	T	A	G	G	T	G	G	T	G	G	G	A	C	G	A	A	C	C
		Guangfengmaliaodou	T	G	T	T	T	T	T	G	C	T	T	G	T	A	G	G	T	G	G	T	G	G	G	A	C	G	G	A	C	C
		Zhonghuang15	C	G	C	G	A	A	T	A	G	T	T	G	T	A	G	G	C	G	G	T	G	G	C	A	C	A	A	T	C	T
		Guixilaidou	C	G	C	G	A	A	T	A	G	T	T	G	T	A	G	G	C	G	G	T	G	G	G	A	C	A	A	A	C	T
		Baoyuheidou	T	G	T	T	T	T	T	G	C	T	T	G	T	A	G	G	C	G	G	T	G	G	G	A	C	G	G	A	C	C
		Shangqiu1099	C	G	C	G	A	A	T	A	G	T	T	G	T	A	G	G	C	G	G	T	G	G	C	A	C	A	A	T	C	T
	Sensitive	Lvhuangdou	T	G	T	T	T	T	T	G	C	T	T	G	T	A	G	G	T	G	G	T	G	G	G	A	C	G	G	A	C	C
		Hongpiyehuangdou	T	G	T	T	T	T	T	G	C	A	G	G	C	G	A	A	T	C	A	C	A	A	G	G	T	A	G	T	C	C
		Shuangjiangzongpidou	C	G	C	G	A	A	T	A	G	T	T	A	T	A	G	G	C	G	G	T	G	G	C	A	C	A	A	T	C	T
		Gaozhouheidou	C	G	C	G	A	A	T	A	G	T	T	G	T	A	G	G	C	G	G	T	G	G	C	A	C	G	A	T	C	T
		Shanghailvyuebai	C	G	C	G	A	A	T	A	G	T	T	G	T	A	G	G	C	G	G	T	G	G	C	A	C	G	A	T	C	T
		Yunxianheidou	C	G	C	G	A	A	T	A	G	T	T	G	T	A	G	G	T	G	G	T	G	G	C	A	C	G	A	T	C	T
		Yantianqingpidou	C	G	C	G	A	A	T	A	G	T	T	G	T	A	G	G	C	G	G	T	G	G	C	A	C	G	A	T	C	T
		Jiangyinheidou	T	G	T	T	T	T	T	G	C	T	T	G	T	A	G	G	T	G	G	T	G	G	G	A	C	G	G	A	C	C
		Linhedafenqing	T	G	T	T	T	T	T	G	C	T	T	G	T	A	G	G	T	G	G	T	G	G	G	A	C	G	G	A	C	C
		Chuandou8	C	G	T	T	T	T	T	G	C	T	T	A	T	A	G	G	T	G	G	T	G	G	G	A	C	G	A	A	C	C
		Han332	T	G	C	T	T	T	T	G	C	T	T	G	T	A	G	G	T	G	G	T	G	G	G	A	C	G	G	A	C	C
		Lingchuanshiyuehuang	C	G	C	G	A	A	T	A	G	T	T	G	T	A	G	G	T	G	G	T	G	G	C	A	C	G	A	T	C	T
		Tianlong1	T	G	C	T	T	T	T	G	C	T	T	A	T	A	G	G	T	G	G	T	G	G	G	A	C	G	G	A	C	C
		Xudou2	T	G	T	T	T	T	T	G	C	T	T	G	T	A	G	G	T	G	G	T	G	G	G	A	C	G	G	A	C	C
		ZhengYang	T	G	T	T	T	T	T	G	C	T	T	A	T	A	G	G	T	G	G	T	G	G	G	A	C	G	G	A	C	C

Supplementary Figure 8. Schematic graph shows the positions and polymorphisms of SNPs in the 2-kb promoter region of soybean *NHX6* gene.

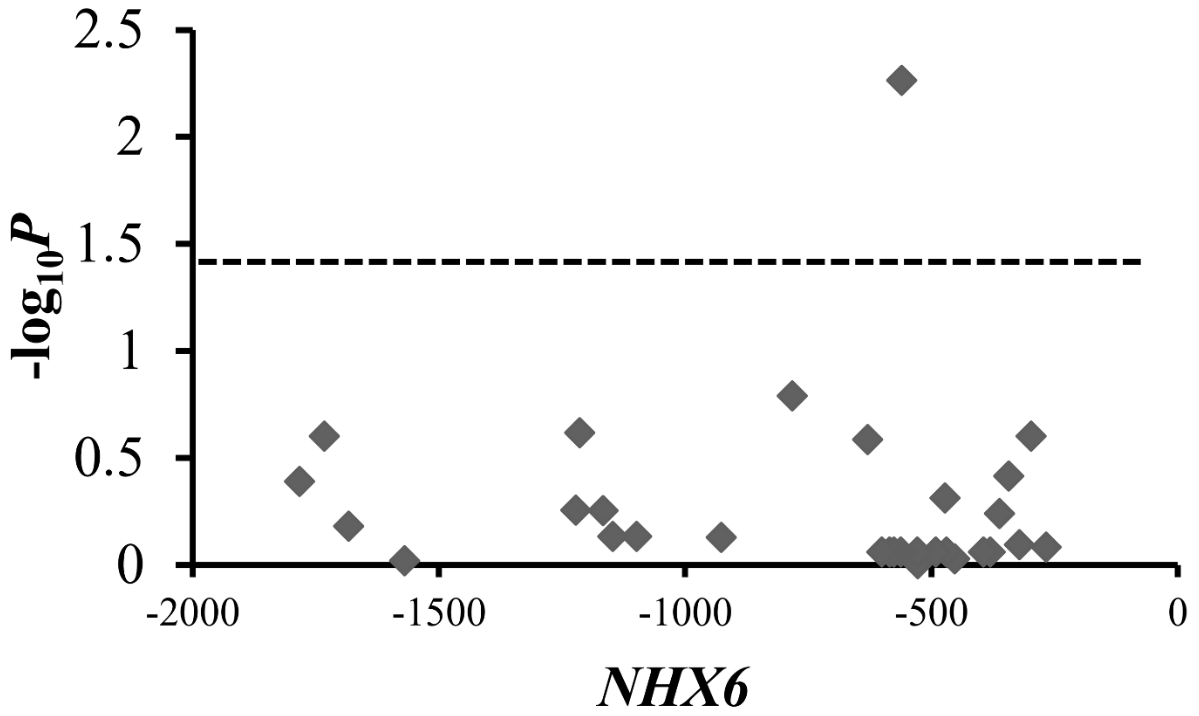

Supplementary Figure 9. Association of sodic tolerance rating (STR) with the SNPs in soybean *NHX6* promoter region. The x-axis represents the physical position of each SNP in base pairs (bp) upstream of the start codon of *GmNHX6* gene. The black dotted line represents the significance threshold of significant associations by mixed model, which is $-\log_{10}(1/30) = 1.48$.

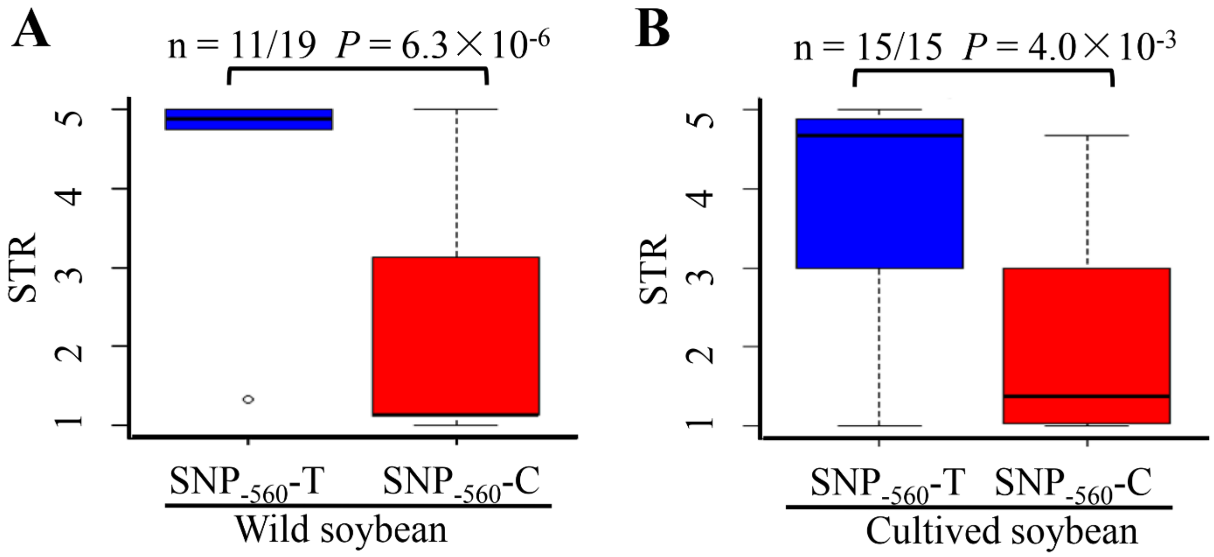

Supplementary Figure 10. Boxplots of sodic tolerance rating (STR) for the two alleles of SNP$_{-560}$ in wild (A) and cultivated (B) soybean accessions.

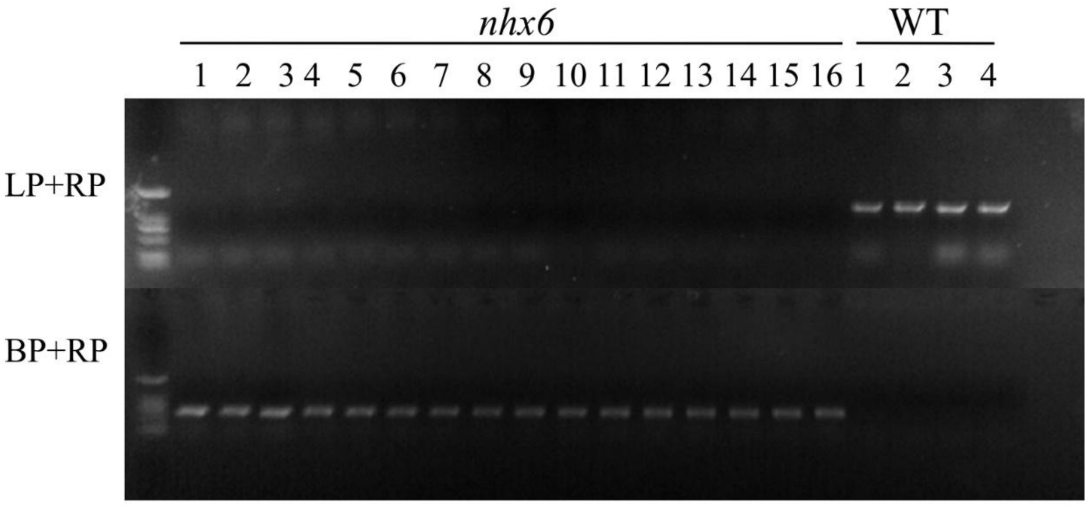

Supplementary Figure 11. Verification of homozygous *nhx6* mutant lines by the method of three primers. LB: Left border primer of the T-DNA insertion, TCAAACAGGATTTTCGCCTGCT, RP: Right genomic primer, TTCCGTCAACAAACATTAGCC, LP: Left genomic primer, TCCTCGGAAAGGAAGAAGAAG.

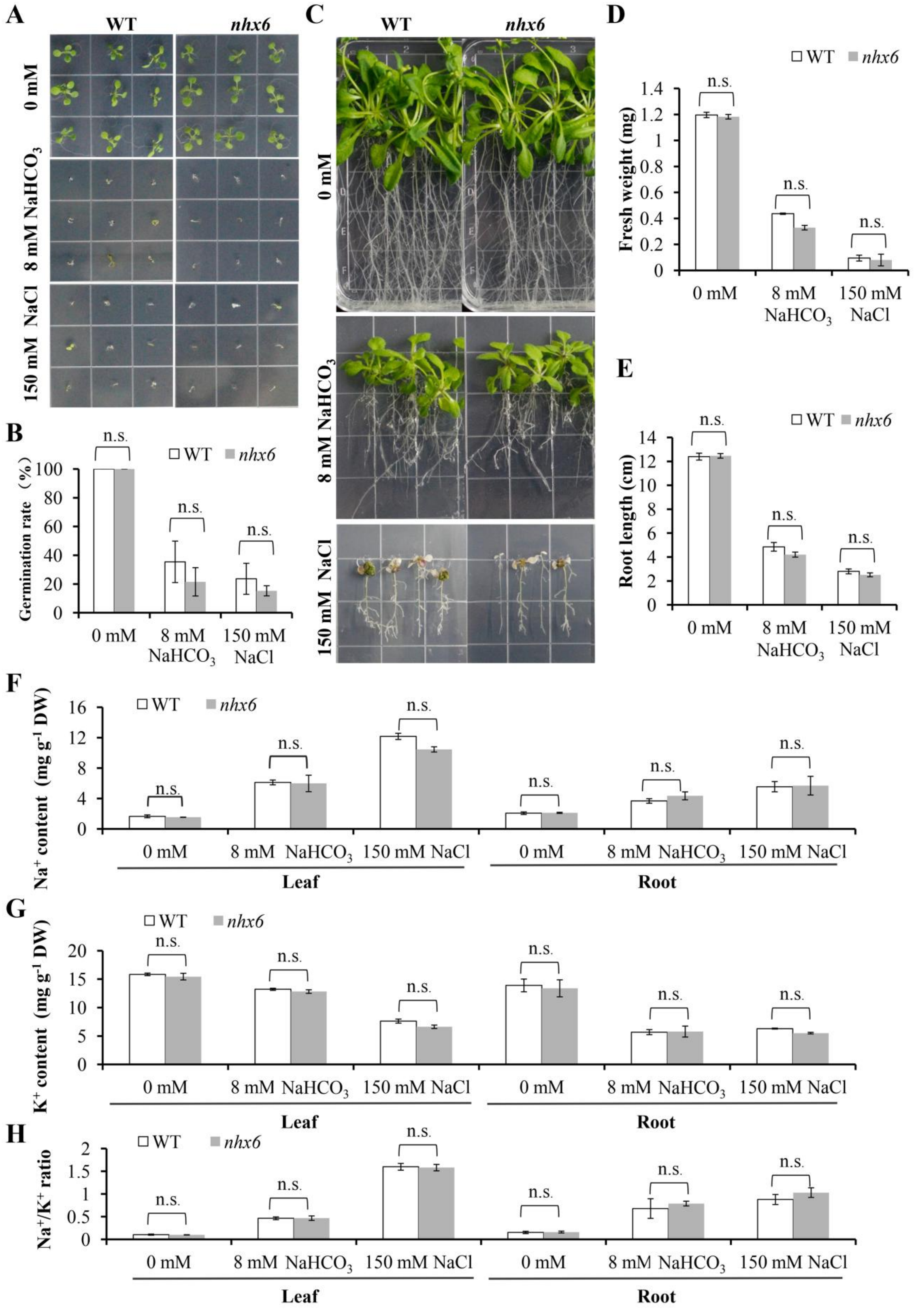

Supplementary Figure 12. Phenotypes of *Arabidopsis nhx6* mutant and wild type in response to $NaHCO_3$ and NaCl treatment. (A) Seed germination assay of different *Arabidopsis* lines on 1/2 MS

medium or media supplied with 8 mM $NaHCO_3$ or 150 mM NaCl, respectively. WT: wild type. **(B)** Seed germination rates of different *Arabidopsis* lines. **(C)** Phenotypes of *Arabidopsis* lines on 1/2 MS medium or media with 8 mM $NaHCO_3$ or 150 mM NaCl, respectively. **(D, E)** Fresh weight and root length of *Arabidopsis* lines. **(F)** Na^+ content, **(G)** K^+ content, **(H)** Na^+/K^+ ratio in the leaves and roots of each line receiving different treatments for 10 days, respectively. Data represents the mean ± standard deviation of three biological replications and each repeat contained four independent plants per line for each treatment (n = 12). n.s.: no significant difference by two-tailed Student's t tests.

```
                                                                                  dOCT
-2000(M)   TTTCACTCTGCACTGTAAGCCGGTGAAGTGCAGTAATTGTGGTAAGCTTTAGTTTTTATCTCTCGGATCTTATACATAAGTTCCTGCATTTACCAAGGGA
-2000(ZY)  TTTCACTCTGCACTGTAAGCCGGTGAAGTGCAGTAATTGTGGTAAGCTTTAGTTTTTATCTCTCGGATCTTATACATAAGTTCCTGCATTTACCAAGGGA

-1900(M)   ATGTGCAGATAAAACGCATACAACGCAACACAAAGGTTCAAAACGTTGAAGAAGGAAAATAAACAGTATAAACTACTTCACCCACGCACATACCTCGACG
-1900(ZY)  ATGTGCAGATAAAACGCATACAACGCAACACAAAGGTTCAAAACGTTGAAGAAGGAAAATAAACAGTATAAACTACTTCACCCACGCACATACCTCGACG
             ABRE                                                                                TGACG-motif   ARE
-1800(M)   GCACGTGTGGCAGCAGCGAGATCCTCCTTCGCCTTAGTTCCTGAGTTGCTCTGGAATCAGAGAATCGGAGCCATCAGAGAGCGAAATTGACGGAACAAAA
-1800(ZY)  GCACGTGTGGCAGCAGCGAGATCCTCCTTCGCCTTAGTTCCTGAGTTGCTCTGGAATCAGAGAATCGGAGCCATCAGAGAGCGAAATTGACGGAACAAAA

-1700(M)   CCAAATCAATGAAATCACTGGACGCTAATTGAAGAGATATTAACACCTGCTGGCGAACGGCGGCGAGAATTCCGGCATCGACGGTGCGAATCTCGTTCTG
                            *
-1700(ZY)  CCAAATCAATGAAATCATTGGACGCTAATTGAAGAGATATTAACACCTGCTGGCGAACGGCGGCGAGAATTCCGGCATCGACGGTGCGAATCTCGTTCTG
                                                                 MYB
-1600(M)   GATTTTCTGCATCAGCGGCTCCACGCCGGAGAGAGACGCCTCTGCGTAACAACAGGCAAATTTGAGAGAAGTTAAGGTTTCGATTAGAAACGCTTGCGAT
                                        *
-1600(ZY)  GATTTTCTGCATCAGCGGCTCCACGCCGGATAGAGACGCCTCTGCGTAACAACAGGCAAATTTGAGAGAAGTTAAGGTTTCGATTAGAAACGCTTGCGAT
                          MYB recognition site
-1500(M)   TGCGAGTGAGATCGGGAATTGTGGCGTACCGTTGGGGAACATCTGGTTGATGTACTCCAGAGCGCTTGACTTGTCCATTGGTGAGAATGAACGCAATCAC
-1500(ZY)  TGCGAGTGAGATCGGGAATTGTGGCGTACCGTTGGGGAACATCTGGTTGATGTACTCCAGAGCGCTTGACTTGTCCATTGGTGAGAATGAACGCAATCAC

-1400(M)   AAAAGCATGAACCGTGATGGTGATTGTGATTAATGTTCAGATCTCTAAACTTGCGTGTTCGGCACCATTTATGCTCTTCAATTTGTGGCGGATCCAATTG
-1400(ZY)  AAAAGCATGAACCGTGATGGTGATTGTGATTAATGTTCAGATCTCTAAACTTGCGTGTTCGGCACCATTTATGCTCTTCAATTTGTGGCGGATCCAATTG
                       TGACG-motif
-1300(M)   CAGATGTGTGTGCTGTGACTGTGACGCTATTGTTAGTGCTCCTCCTTTTCGTCGTCTTCAACCTCTGGACACTTCTGTAATATTTTATATATATATACTA
                                                                                                  *      *
-1300(ZY)  CAGATGTGTGTGCTGTGACTGTGACGCTATTGTTAGTGCTCCTCCTTTTCGTCGTCTTCAACCTCTGGACACTTCTGTTATATTTTTATATATATATACTA
                                                               ABRE                                  ERE
-1200(M)   CTATTTTATATATGGATGAAAACTAAAATATGTTTTTTACTGAAAAATCACGTAAGTATGAATTTCATTCTTGTAAAACTTTTTATTTAAAATATATCTA
                                                                  *
-1200(ZY)  CTATTTTATATATGGATGAAAACTAAAATATGTTTTTTACTGAAAAATCACGTGAGTATGAATTTCATTCTTGTAAAACTTTTTATTTAAAATATATCTA
                                                                       W box
-1100(M)   GGTTCGATTGATTCTCATGTCAATGTATGATTCTATTTCTTATGTGTCACTCCCTTGTTGACCAAATTCTTGACTACTCTCTTTTTCTCATAATCAAAAC
            *
-1100(ZY)  GCTTCGATTGATTCTCATGTCAATGTATGATTCTATTTCTTATGTGTCACTCCCTTGTTGACCAAATTCTTGACTACTCTCTTTTTCTCATAATCAAAAC

-1000(M)   TTCCCAAACTTGAAAAAAAGAAAGGCCTCAAATTTGCAAATTGATTTAATGCTCCTTCATCATAAATTTGACATATAGTAACAAAAAGAAAAAGGCTTTG
-1000(ZY)  TTCCCAAACTTGAAAAAAAGAAAGGCCTCAAATTTGCAAATTGATTTAATGCTCCTTCATCATAAATTTGACATATAGTAACAAAAAGAAAAAGGCTTTG
                                              Box 4   ERE
-900(M)    GGGTTAGTTTTATAGTTGACTTTTTATTACGCTAAAATTAATATTTAAAATTTTCTTATTATGTATTATTATTTATATTAATTTTAGTTTAGTTGGTTTT
-900(ZY)   GGGTTAGTTTTATAGTTGACTTTTTATTACGCTAAAATTAATATTTAAAATTTTCTTATTATGTATTATTATTTATATTAATTTTAGTTTAGTTGGTTTT
                                                                                                      MYC
-800(M)    TAAAAACATGTTTCATATTAATTTTTTTCTTTATTCCTTTACGTATTTATGACAATATGTTAAGTTGAAAATTAATAAAGGTGAACATGTGTTCCTATAC
-800(ZY)   TAAAAACATGTTTCATATTAATTTTTTT-CTTTATTCCTTTACGTATTTATGACAATATGTTAAGTTGAAAATTAATAAAGGTGAACATGTGTTCCTATAC
                                                             MYB                       AT1-motif    WUN-motif
-700(M)    ATATATTACATAAAGGAAGACCTTCATCTCTCATCATCTAAAAATTAGCTTCAACCATATTAAAATGTTTGGAAAAAAAAATAATTAGTCAAATTACTAT
                                                                                   *
-700(ZY)   ATATATTACATAAAGGAAGACCTTCATCTCTCATCATCTAAAAATTAGCTTCAACCATATTAAAATGTTTGAAAAAAAAAATAATTAGTCAAATTACTAT
                     TGTCAT element   Skn-1_motif   TCATTT element   TTGTCA element
-600(M)    TTCTTTTTTATAATAACATTTATTGTATTGACAATGTTGTCATTTATTAACATTTATTTATCAAATTATATGGATATAACTTTGATCAATTATTACATTG
                                                 *       *
-600(ZY)   TTCTTTTTTATAATAACATTTATTGTATTGACAATGTTGTTATTTATTGACATTTATTTATCAAATTATATGGATATAACTTTGATCAATTATTACATTG
                         Box 4                           GA-motif      TC-rich repeats   TATA
-500(M)    TTGATAAACTTTTCAATTAGATAATATGTAGATTTAAATTAATGATTCAATAGATAAAAATTATATTCTATAACAATTTATAAAATAGTATAATTTGTTT
                                                          *                                         **
-500(ZY)   TTGATAAACTTTTCAATTAGATAATATGTAGATTTAAATTAATGATTGAATAGATAAAAATTATATTCTATAACAATTTATAAAAATGTATAATTTGTTT
                           Box 4     ABRE                 Box 4                    Box 4
-400(M)    AAGAAATATAATTCAAATTCATTTAATATCTTATTCACATAGATTAAAATCAATCTAATATA AATGTTTTTAAATATATTAATATATGAATATTTTGATT
                                           *                  *                 *
-400(ZY)   AAGAAATATAATTCAAATTCATTTAATATCTTATTCACGTAGATTAAAATCAATCTAGTATAAATGTTTTTAAATATATAAATATATGAATATTTTGATT
                                                                                              AT-rich element
                                                           (ZY)
-300(M)    ATCTATTTGTAGTTTAATTTTAATAAAATTTGATATAAACGATCTTAATAATTTATAAATATAATTTAAGGTGGATTAATAAAAATCAAATTTTATAATA
                                              *    *                             *
-300(ZY)   ATCTATTTGTAGTTTAATTTTAATAAAATTTGACATAAATGATCTTAATAATTTATAAATATAATTTAAGGCGGATTAATAAAAATCAAATTTTATAATA
            Box 4     Box 4                                                     ARE           ATCT-motif
-200(M)    TTAATATATTTAATATTCCGGAGTCCAAGAGGTGAGAAGAACAGAGAAACAGAATTGAAAAACTCACAAACCAGATAGAGAGAATCTAATCAGAACAGAG
-200(ZY)   TTAATATATTTAATATTCCGGAGTCCAAGAGGTGAGAAGAACAGAGAAACAGAATTGAAAAACTCACAAACCAGATAGAGAGAATCTAATCAGAACAGAG
                                                                            GCN4_motif
-100(M)    TTCTGTTCCTTTTCTTCTGATTCCCGAAGAACTCAACGATCCAATCGCAACCGGAAGCAATCGATCAGTGAGTCAATTGCTTCCGGCAAGGAGAACAATA
-100(ZY)   TTCTGTTCCTTTTCTTCTGATTCCCGAAGAACTCAACGATCCAATCGCAACCGGAAGCAATCGATCAGTGAGTCAATTGCTTCCGGCAAGGAGAACAATA
```

*:SNP site.   M: Meng8206.   ZY: ZhengYang.

Supplementary Figure 13. The *cis*-element analysis of the 2-kb promoter region upstream of start codon ATG of *GmNHX6* from soybean varieties of M8206 (M) and ZY.

Supplementary Table 1. List of 60 soybean accessions with extreme alkaline salt tolerance (sodic tolerance rating, STR).

Accession	Category	Average STR	Alkaline salt tolerance
LY01-10	Wild soybean	1.00	Tolerant
LY15-07	Wild soybean	1.00	Tolerant
LY04-02	Wild soybean	1.13	Tolerant
LY04-04	Wild soybean	1.13	Tolerant
LY05-06	Wild soybean	1.13	Tolerant
LY12-01	Wild soybean	1.13	Tolerant
LY14-03	Wild soybean	1.13	Tolerant
LY14-09	Wild soybean	1.13	Tolerant
LY16-06	Wild soybean	1.13	Tolerant
LY01-07	Wild soybean	1.13	Tolerant
LY04-01	Wild soybean	1.25	Tolerant
LY04-03	Wild soybean	1.25	Tolerant
LY07-06	Wild soybean	1.25	Tolerant
LY15-01	Wild soybean	1.25	Tolerant
LY04-06	Wild soybean	1.33	Tolerant
LY06-05	Wild soybean	4.75	Sensitive
LY07-03	Wild soybean	4.75	Sensitive
LY09-09	Wild soybean	4.75	Sensitive
LY10-04	Wild soybean	4.88	Sensitive
LY16-10	Wild soybean	4.88	Sensitive
LY03-05	Wild soybean	5.00	Sensitive
LY05-10	Wild soybean	5.00	Sensitive
LY10-07	Wild soybean	5.00	Sensitive
LY13-09	Wild soybean	5.00	Sensitive
LY15-09	Wild soybean	5.00	Sensitive
LY16-01	Wild soybean	5.00	Sensitive
LY19-01	Wild soybean	5.00	Sensitive
LY21-03	Wild soybean	5.00	Sensitive
LY10-01	Wild soybean	5.00	Sensitive
LY21-01	Wild soybean	5.00	Sensitive
Meng8206	Cultivated soybean	1.00	Tolerant
Wuheqihuangdou	Cultivated soybean	1.00	Tolerant
Williams82	Cultivated soybean	1.00	Tolerant
Zhongdou8	Cultivated soybean	1.00	Tolerant

Accession	Category	Average STR	Alkaline salt tolerance
Jindou6	Cultivated soybean	1.00	Tolerant
Erwangdou	Cultivated soybean	1.00	Tolerant
Yudou22	Cultivated soybean	1.05	Tolerant
Pingbuxiaohuangdou	Cultivated soybean	1.20	Tolerant
Gongdou2	Cultivated soybean	1.16	Tolerant
Xudou8	Cultivated soybean	1.44	Tolerant
Guangfengmaliaodou	Cultivated soybean	1.33	Tolerant
Zhuanghuang15	Cultivated soybean	1.37	Tolerant
Guixilaidou	Cultivated soybean	1.44	Tolerant
Baoyuheidou	Cultivated soybean	1.44	Tolerant
Shangqiu1099	Cultivated soybean	1.44	Tolerant
Lvhuangdou	Cultivated soybean	4.55	Sensitive
Hongpiyehuangdou	Cultivated soybean	4.56	Sensitive
Shuangjiangzongpidou	Cultivated soybean	4.56	Sensitive
Gaozhouheidou	Cultivated soybean	4.56	Sensitive
Shanghailvyuebai	Cultivated soybean	4.56	Sensitive
Yunxianheidou	Cultivated soybean	4.56	Sensitive
Yantianqingpidou	Cultivated soybean	4.67	Sensitive
Jiangyinheidou	Cultivated soybean	4.67	Sensitive
Linhedafenqing	Cultivated soybean	4.70	Sensitive
Chuandou8	Cultivated soybean	4.78	Sensitive
Han332	Cultivated soybean	4.89	Sensitive
Lingchuanshiyuehuang	Cultivated soybean	4.89	Sensitive
Tianlong1	Cultivated soybean	5.00	Sensitive
Xudou2	Cultivated soybean	5.00	Sensitive
ZhengYang	Cultivated soybean	5.00	Sensitive

Supplementary Table 2. Primers used for qRT-PCR, gene cloning, and vector construction.

**Primer sets**	**Forward primer sequences**	**Reverse primer sequences**	**Purpose**	**Amplification Efficiency (Mean ±SD)**
q- *GmNHX6*	TGCTGCCTCTTTTACTGCATTAGAT	ACCATGAAGTTCCGATCTTGTAGAA	qRT-PCR	1.01 ±0.01
GmUKN1	TGGTGCTGCCGCTATTTACTG	GGTGGAAGGAACTGCTAACAATC	qRT-PCR	0.99 ±0.02
AtACTIN7	CCTCAGCACCTTCCAACAGATG	AACTCACCACCACGAACCAGAT	qRT-PCR	1.02 ±0.01
c-*GmNHX6*	GTGAGTCAATTGCTTCCGGC	GCACAAGTAATGCGCACCAA	Gene cloning	
c-*GmNHX6*-promoter	CTTCACCCACGCACATACCT	GAGGACGAAGGACAAGACCAG	Promoter cloning	
v-pBinGFP4-*GmNHX6*	TCCCCCGGGATGAC CTCGGAGTTAGAAAT	CGCGGATCCTGATGAA TAGTGGTTCTGACC	Vector construction	
v-pCAMBIA3301-*GmNHX6*	tctagaggatctcgaggcgcgcc ATGACCTCGGAGTTAGAAAT	attcgagctcactagttaattaa TGATGAATAGTGGTTCTGACC	Vector construction	
v-pAN580-*GmNHX6*	CTAGCTAGCATGACC TCGGAGTTAGAAATTTCTC	CCCCCCGGGTGATGA ATAGTGGTTCTGAC	Vector construction	
c-GmNHX6-promoter-LUC	ctatagggcgaattgggtacc CTTCACCCACGCACATACCTC	atcgataccgtcgacctcgag TATTGTTCTCCTTGCCGGAAGC	Vector construction	

Supplementary Table 3. The summary of the Ct values of the reference gene GmUKN1 for qRT-PCR across all samples in this study.

Minimum	Maximum	Mean	Standard Deviation
19.99	20.99	20.53	0.25

Note: A total of 540 Ct values from 60 samples × 3 biological replications × 3 technical repeats.

Supplementary Table 4. Regional association study of the alkaline salt tolerance.

SNP (Chr_bp)	Position[a]	Allele (major: minor)	MAF[b] (%)	*P*-value (MLM[c])
9_1430060	-267	T:C	38.33%	0.7210
9_1430091	-298	C:T	11.67%	0.2249
9_1430114	-321	T:A	35.00%	0.7803
9_1430136	-343	A:G	38.33%	0.4516
9_1430155	-362	A:G	40.00%	0.7390
9_1430174	-381	C:T	10.00%	0.8274
9_1430188	-395	A:G	10.00%	0.8274
9_1430246	-453	C:G	40.00%	0.8948
9_1430263	-470	G:A	10.00%	0.8274
9_1430266	-473	G:A	11.67%	0.5628
9_1430284	-491	T:C	10.00%	0.8274
9_1430321	-528	G:A	15.00%	0.9868
9_1430322	-529	G:C	10.00%	0.8274
9_1430353	-560	C:T	43.33%	0.0055
9_1430355	-562	G:A	10.00%	0.8274
9_1430369	-576	G:A	10.00%	0.8274
9_1430378	-585	A:G	10.00%	0.8274
9_1430394	-601	T:C	10.00%	0.8274
9_1430423	-630	G:A	6.67%	0.2558
9_1430575	-782	T:G	13.33%	0.1598
9_1430720	-927	T:A	23.33%	0.8753
9_1430892	-1099	G:C	50.00%	0.6015
9_1430940	-1147	A:G	50.00%	0.6015
9_1430960	-1167	T:A	10.00%	0.5348
9_1431007	-1214	A:T	35.00%	0.2001
9_1431015	-1222	A:T	46.67%	0.5750
9_1431363	-1570	G:T	35.00%	0.9620
9_1431476	-1683	C:T	36.67%	0.6813
9_1431526	-1733	G:A	11.67%	0.2249
9_1431576	-1783	C:T	25.00%	0.4274

[a]Position: position from the start codon; [b]MAF: minor allele frequency; [c] MLM: Mixed linear model.
